# Adaptation to prolonged neuromodulation in cortical cultures: an invariable return to network synchrony

**DOI:** 10.1186/s12915-014-0083-3

**Published:** 2014-10-23

**Authors:** Maya Kaufman, Sebastian Reinartz, Noam E Ziv

**Affiliations:** Department of Physiology and Biophysics and Rappaport Institute, Technion Faculty of Medicine, and Network Biology Research Laboratories, Lorry Lokey Center for Life Sciences & Engineering, Fishbach Building, Haifa, 32000 Israel

**Keywords:** Neuromodulators, Synchrony, Acetylcholine, Closed-loop, Adaptation, Cultured neuronal networks, Multielectrode arrays

## Abstract

**Background:**

Prolonged neuromodulatory regimes, such as those critically involved in promoting arousal and suppressing sleep-associated synchronous activity patterns, might be expected to trigger adaptation processes and, consequently, a decline in neuromodulator-driven effects. This possibility, however, has rarely been addressed.

**Results:**

Using networks of cultured cortical neurons, acetylcholine microinjections and a novel closed-loop ‘synchrony-clamp’ system, we found that acetylcholine pulses strongly suppressed network synchrony. Over the course of many hours, however, synchrony invariably reemerged, even when feedback was used to compensate for declining cholinergic efficacy. Network synchrony also reemerged following its initial suppression by noradrenaline, but this did not occlude the suppression of synchrony or its gradual reemergence following subsequent cholinergic input. Importantly, cholinergic efficacy could be restored and preserved over extended time scales by periodically withdrawing cholinergic input.

**Conclusions:**

These findings indicate that the capacity of neuromodulators to suppress network synchrony is constrained by slow-acting, reactive processes. A multiplicity of neuromodulators and ultimately neuromodulator withdrawal periods might thus be necessary to cope with an inevitable reemergence of network synchrony.

**Electronic supplementary material:**

The online version of this article (doi:10.1186/s12915-014-0083-3) contains supplementary material, which is available to authorized users.

## Background

Neuronal networks are strongly influenced by neuromodulatory systems; consequently, the properties and dynamics of particular networks can vary enormously depending on the levels, timing and composition of neuromodulatory influences [1]. While such influences often vary on short time scales, neurons are also influenced by changes in neuromodulatory input over relatively long time scales. In many biological systems, prolonged exposure to agonists is associated with adaptation to those substances or their effects. Neurons, in particular, are known to adapt or react homeostatically to changes in their input levels or in their milieu (reviewed in [2-4]; see also [5,6]), raising the possibility that prolonged neuromodulation will be associated with some recovery of the affected properties. At present, however, the question of adaptive or homeostatic reactivity to long-term neuromodulation has rarely been addressed.

Some of the most important long-term neuromodulatory processes in the mammalian brain are those that regulate a striking form of cortical synchrony known as ‘slow oscillations’, ‘slow wave’ or ‘slow rhythmic’ activity. This activity pattern is characterized by transitions between periods of neuronal discharges (‘on’ periods) and periods of near-complete quiescence (‘off’ periods) which occur in remarkably synchronous fashion in large cortical domains. Macroscopically, these synchronous transitions appear in electroencephalogram (EEG) recordings as low frequency, high amplitude waves (for example, [7-15]; reviewed in [16]). At the single neuron level, these transitions typically coincide with transitions between relatively depolarized membrane potentials (‘up’ states), which are often accompanied by action potential firing and strong hyperpolarizations (‘down’ states) during which neurons are silent. Following the lead of Harris and Thiele [17] we will refer to these network-wide, synchronous transitions between ‘on’ and ‘off’ periods as ‘synchrony’. Note that in this context synchrony does *not* refer to the degree to which multiple neurons fire action potentials simultaneously at millisecond time precision.

In the intact brain, synchrony as defined above is strongly regulated by brainstem and basal forebrain noradrenergic and cholinergic neurons which project to widespread cortical regions [16,18-20]. The activation of these neuromodulatory systems strongly suppresses network synchrony and promotes asynchronous activity patterns typical of aroused and attentive behavioral states. In contrast, reduced activity of these systems, which occurs mainly during periods of NREM (non rapid eye movement) sleep, is associated with the prominent appearance of network synchrony as defined above. Importantly, however, neither these forms of network synchrony nor their modulation by acetylcholine (ACh) and noradrenaline (NA) are limited to the intact cortex, as similar activity patterns occur in brain slabs [21], acute and organotypic cortical preparations (for example, [22-26]) and even in networks of dissociated cortical neurons (for example, [27-37]; reviewed in [38]).

Where networks of dissociated cortical neurons in culture are concerned, synchrony takes the form of network-wide bursting activity which lasts for several hundreds of milliseconds, separated by longer periods (1 to 10 seconds) of near-complete quiescence or sparse, asynchronous action potentials [27-37]. These network-wide bursts are less frequent and more stereotyped as compared to those observed in the intact brain, which might be expected given the smaller size and lower connection density of these networks as well as the lack of reentrant pathways [21,39-41]). Moreover, it has been suggested that the degree of synchrony in these and other *in vitro* preparations is exacerbated by various homeostatic responses to deafferentation, resulting in activity forms that share some similarities with seizure-related paroxysmal activity (as indicated by *in vivo* deafferentation studies [42,43]). Yet, while the forms of synchrony observed *in vitro* differ in many respects from those associated with low arousal levels in the intact brain, their underlying biophysical mechanisms share important similarities. Both *in vivo* [10,15,17,21,44] and *in vitro* [27,33,34,37,45-47] experiments, as well as modeling studies [21,39,40,48,49], indicate that these forms of synchrony are not imposed by some external circuitry, global inhibition or pacemaker cells, but probably arise from the interplay of spontaneous synaptic activity, nonlinear neuronal recruitment cascades, refractoriness and network wide synaptic depression (summarized in [17]), effectively giving rise to a *default* activity mode, as it has been referred to [35,36] (see also [39]). Furthermore, and in full concordance with their activities *in vivo* [44,50-61], cholinergic and adrenergic agonists suppress network synchrony in cell culture and slice preparations, shifting spontaneous activity away from this ‘default’ mode towards desynchronized, tonic firing modes [35,36,62-65]. Thus, while synchrony in networks of cultured cortical neurons does not fully replicate the forms of synchrony related to low neuromodulatory tone in the intact brain, the similarities in underlying mechanisms and the comparable effects of neuromodulation suggest that this preparation is a useful model system for studying relationships between prolonged neuromodulation and network synchrony.

As mentioned above, the tendency of neurons and neuronal networks to adapt or react homeostatically raises the possibility that prolonged neuromodulation will be associated with some reactive adaptation over long time scales. Indeed, in a prior study [35] we found that the chronic, prolonged (many hour) exposure of networks of cultured cortical neurons to a cholinergic agonist is associated with the gradual growth of excitatory synapses and, intriguingly, to the gradual reemergence of synchrony (see also [36]). If neuronal networks adapt to neuromodulatory input, it might be asked how the necessity to suppress network synchrony is ultimately addressed, in particular given that this activity form seems to be incompatible with attentive states and arousal [66,67]. Conceivably, the contradiction between this necessity and diminishing neuromodulator efficacy might have been resolved by adjusting neuromodulatory input to match instantaneous neuromodulator efficacy. Alternatively, this contradiction might have been alleviated by the existence of multiple neuromodulatory systems [18] that exert similar effects but employ different cellular mechanisms. If, however, neither of these routes resolve the need to suppress synchrony, periodic neuromodulator withdrawal periods (such as those which occur during NREM sleep periods) might be ultimately required. To date, however, none of these possibilities have been explored or addressed experimentally.

Here we used a system based on networks of cultured cortical neurons, ACh microinjections and a novel closed-loop ‘synchrony-clamp’ to address these questions. Specifically, we examined the capacity of both fixed and feedback-based adjusted cholinergic input to suppress network synchrony continually as defined above. We then examined the ability of multiple neuromodulators to suppress network synchrony continually. Finally we examined the possibility that cholinergic neuromodulatory efficacy might be preserved on extended time scales by periodically withdrawing cholinergic input.

## Results

### Rational and experimental approach

**Figure 1:**
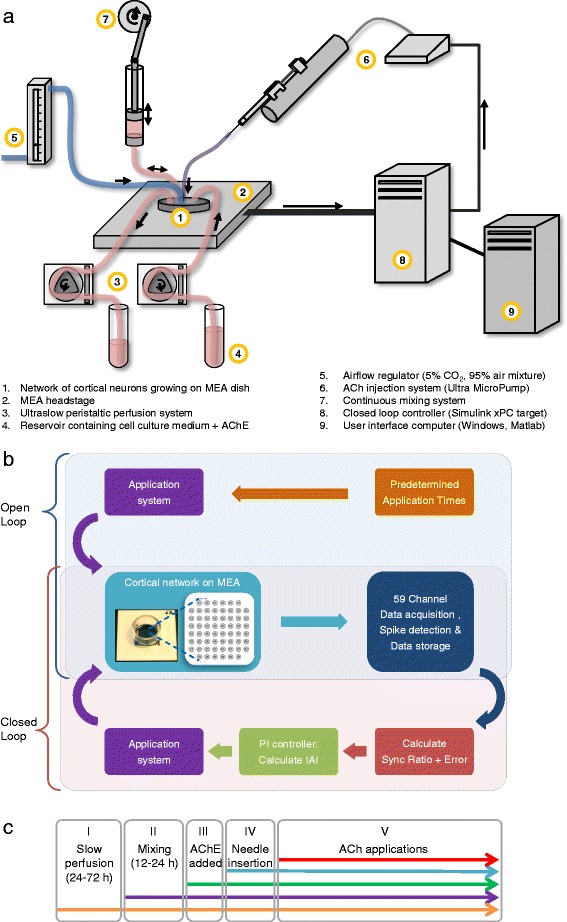
**Setup used to examine long-term effects of ACh applications on network synchrony in open-and closed-loop regimes. a)** Schematic illustration of the experimental system used to record from neurons growing on MEA substrates, maintain their viability, and apply ACh pulses at predetermined rates or at rates adjusted online to maintain network synchrony at predefined levels. **b)** Schematic illustration of open- and closed-loop experimental protocols. Note that in open-loop experiments ACh application timings are predefined, whereas in closed-loop experiments, ACh application timings are determined online according to instantaneous Sync Ratio values calculated by the real-time controller (item 8 in a, see Additional file 3: Figure S3 for an explanation of the Sync Ratio measure). **c)** Preparatory and experimental phases. Each experiment was preceded by the following preparatory phases: Phase I, slow perfusion (at least 24 hours); Phase II, activation of continuous mixing system (12 to 24 hours); Phase III, addition of AChE (0.1 U/ml) to the MEA dish and perfusion media reservoir (30 minutes, see Additional file 7: Figure S6 for the importance of AChE addition); Phase IV, insertion of ACh application needle into the MEA dish; and Phase V, experiment (open or closed loop). Note that the experimental component added in each phase was in effect from that moment until the end of the experiment. See Methods for further details. ACh, acetylcholine; AChE, acetylcholine esterase; MEA, multielectrode array.

To examine relationships between prolonged cholinergic input and network synchrony we developed an experimental system which allowed us to tightly control and manipulate cholinergic input, measure its effects on network activity and synchrony, and assay changes in its capacity to suppress synchrony, with the latter serving as a measure of adaptive or homeostatic reactions occurring in the same networks. This system, shown schematically in Figure 1, is based on primary cultures of dissociated rat cortical neurons growing on substrate integrated multielectrode arrays (MEAs), a microinjection system used to apply minute ACh solution volumes in the form of brief pulses, and a controller used to apply ACh at fixed intervals (open-loop experiments) or at intervals modified online as required to ‘clamp’ synchrony at fixed, low levels (closed-loop experiments). To terminate ACh signaling after each application and avoid ACh buildup in the media, acetylcholine esterase (AChE) was added to the network cell culture media (and perfusion system), assuring continuous and efficient ACh enzymatic breakdown [68]. ACh injections were delivered via a needle immersed in the culture media, hovering about 1 mm above the neuronal network (Figure 1a, item 6), whereas a slow continuous mixing system was used to accelerate the dilution and distribution of the applied ACh and facilitate its subsequent enzymatic breakdown (Figure 1a, item 7). In addition, the system also included provisions for optimal environmental conditions (a slow perfusion system, a stream of a sterile gas mixture and heating devices, Figure 1a, items 3 to 5), providing a stable environment of 37°C, 5% CO_2_ and one to two media replacements/day, resulting in experiments which were effectively open-ended [69].

### Pulsed ACh applications effectively suppress network synchrony, but synchrony eventually reemerges

**Figure 2:**
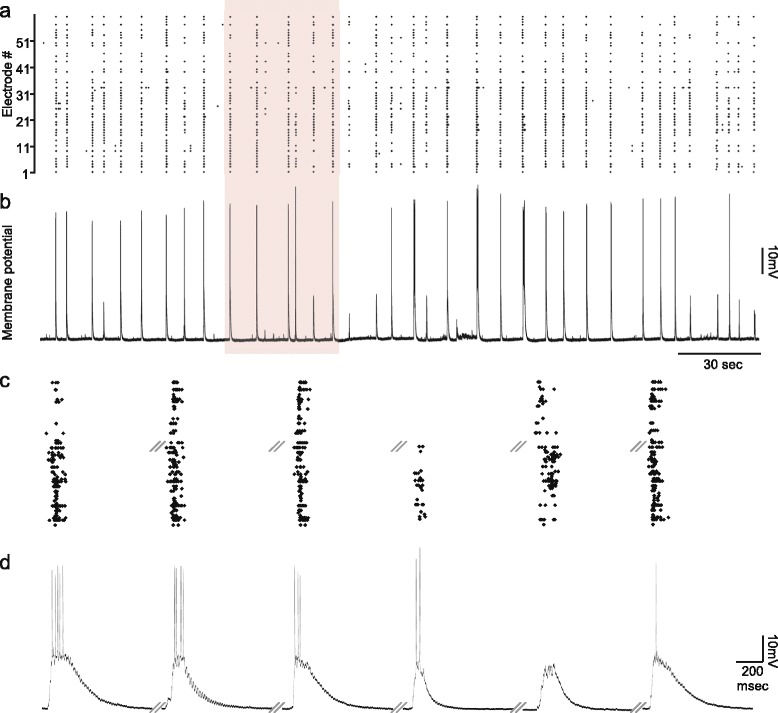
**Synchronous bursts and quiescent periods are well correlated with neuronal membrane potential depolarization (‘up’ states) and hyperpolarization (‘down’ states).** Combined intracellular whole-cell patch clamp and extracellular recordings from neurons growing on MEA substrates are shown. **a)** A raster plot depicting 260 seconds of network activity recorded from 59 extracellular electrodes. Each point represents an action potential recorded from that electrode. **b)** Concomitant intracellular recording of membrane potential from one neuron in the network over the same period. An excellent correspondence between membrane potential depolarizations and network-wide bursting is evident. **c)** Six events from the pink rectangle in a, shown at higher temporal resolution. **d)** Corresponding intracellular voltage traces for the same events shown in c. Note that each network burst is associated with strong membrane depolarization, usually crowned by one or more action potentials. These events are remarkably similar to cortical ‘up’ states observed in sleeping and anesthetized animals. Each trace in c and d is one second long. Backslashes denote omitted time periods. The initial membrane potential in b and d was approximately -55 mV. MEA, multielectrode array.

Under baseline conditions, and in agreement with many reports [27,30-36,70], spontaneous activity in the cortical networks used here occurs as periods of synchronous, network-wide bursting activity which lasts for several hundreds of milliseconds, separated by longer periods (1 to 10 seconds) of near-complete quiescence or sparse, asynchronous action potentials (Figure 2a). As mentioned in the introduction, these network activity patterns share many similarities with the forms of synchrony observed *in vivo* under regimes of low neuromodulatory levels. As shown in Figure 2b, this similarity also extends to the single neuron level: intracellular whole-cell recordings from individual neurons (six neurons in three networks) performed concomitantly with extracellular recordings from the 59 electrodes of the MEAs invariably showed that periods of network-wide bursting activity were tightly correlated with neuronal membrane potential depolarizations, often accompanied by the firing of action potentials, whereas network quiescence periods were associated with deep and constant membrane hyperpolarizations (Figure 2a,b). Closer examination (Figure 2d) revealed that these depolarizations and hyperpolarizations exhibited a marked resemblance to ‘up’ and ‘down’ states recorded *in vivo* (compare, for example, with [71]), as did their tight temporal correlation with network ‘on’ and ‘off’ periods (Figure 2c), although their occurrence was less frequent (see Background).

Our first goal was to examine how the forms of network synchrony described above are affected by cholinergic input delivered at a fixed rate. To that end, we performed the following experiments: cortical networks growing on MEA dishes (maintained in culture for at least 17 days) were mounted on the MEA headstage and provided with optimal environmental conditions as described above. After several preparatory phases (Figure 1c, phases I-IV; see Additional file 1: Figure S1 and Methods), including the activation of the slow continuous mixing system mentioned above (Figure 1a, item 7), the experiments were started by initiating automated ACh applications at fixed intervals (Figure 1c, phase V), that is, in an ‘open-loop’ regime as illustrated in Figure 1b. ACh applications consisted of minute volumes (1 μl) of concentrated ACh solution (20 mM) briefly injected at five minute intervals into the media bathing the cortical networks, which, as mentioned above, contained AChE and was continually mixed. Ignoring ACh breakdown, a single, well-mixed ACh application would be expected to elevate ACh concentration to a final value of 10 μM. The particular experimental profile used here was chosen after an extensive series of preliminary experiments in which we explored a wide range of ACh concentrations and application rates, settling on this regime as a compromise between the desire to mimic physiological profiles of cholinergic neuromodulation and the constraints imposed by the finite volumes of the injection syringe and MEA dish, mixing rates, synchrony stability and the time course of synchrony recovery.

**Figure 3:**
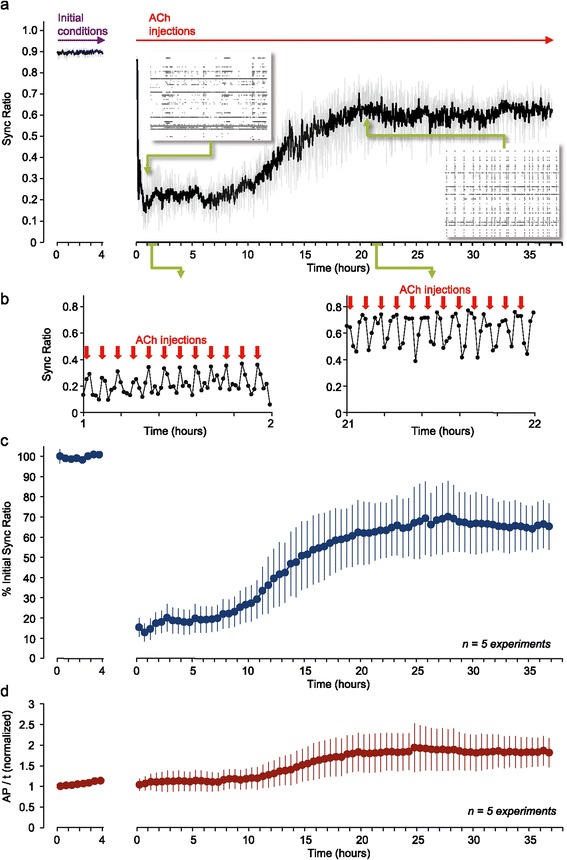
**Synchronous activity reemerges following semi-phasic, periodic ACh applications. a)** Sync Ratio over time in one experiment (grey; same data after smoothing with a five-point kernel is shown in black), performed in an open-loop regime. ACh pulses were delivered once every five minutes, starting at t =5 minutes. Note the high values of the Sync Ratio in the period preceding ACh applications (initial conditions, obtained during the period defined as phase II in Figure 1c; see also Additional file 1: Figure S1) and the huge reduction in Sync Ratio values once ACh applications were initiated. Insets: examples of one-minute raster plots from early (left) and late (right) stages of the experiment. **b)** Enlarged one-hour sections of the plot shown in a from early (left) and late (right) stages of the experiment. Red arrows denote application times. **c)** Changes in Sync Ratio over time in five separate experiments similar to the experiment shown in a. For each experiment, Sync Ratio values, averaged over 30 minutes, were normalized to the average Sync Ratio measured during the 4 hour period preceding ACh application (initial conditions). Average ± SEM for five experiments. **d)** Total firing rates measured from all electrodes, averaged over 30 minute intervals, normalized for each experiment to the average firing rate measured during the 4 hour period preceding ACh application (Initial conditions). Note that the initiation of ACh applications was not associated with changes in overall firing rates. Average ± SEM for five experiments. ACh, acetylcholine; SEM, standard error of the mean.

One fixed input rate experiment is shown in Figure 3a,b. As shown here, prior to ACh applications synchrony levels were high and quite stable (see also Additional file 1: Figure S1 and Additional file 2: Figure S2). The initiation of ACh applications (starting exactly five minutes after the beginning of the recording phase shown) had major effects on the characteristics of spontaneous activity, the most obvious being the replacement of stereotyped, network-wide alternating periods of activity and silence with much more diverse activity forms (compare the raster plots of Figure 2a with Figure 3a and Additional file 1: Figure S1 and Additional file 3: Figure S3). These ranged from near continuous tonic firing, through intermittent tonic firing, through sporadic firing to near silence. Importantly, and in excellent agreement with prior *in vitro* [35,62-65] and *in vivo* [50,57,61] studies, the network-wide synchrony of neural activity was strongly reduced. To quantify the effects of ACh on network synchrony we developed a robust measure that was termed the ‘Sync Ratio’ which ranges from 0 to 1 (completely asynchronous to completely synchronous activity, respectively; see Methods and Additional file 3: Figure S3 for a full explanation of this measure’s rational and derivation). In this experiment, the Sync Ratio was reduced from approximately 0.9 (mixing system active, no exposure to ACh; Figure 1c, phase II) to approximately 0.2. In general, each ACh application was associated with a transient decrease in the Sync Ratio which then partially recovered over the next few minutes (Figure 3b). As applications were delivered at five minute intervals, network synchrony did not have sufficient time to recover fully. Importantly, after about 10 hours, Sync Ratio values started to gradually increase, finally settling on an intermediate Sync Ratio value after about 20 hours (Figure 3a, note also right-hand raster plot). This reemergence of synchrony occurred in spite of the fact that almost every ACh application was still partially effective as shown in Figure 3b.

The average evolution of the Sync Ratio (normalized to the average Sync Ratio during the four-hour pre ACh applications period) for five fixed input rate (open-loop) experiments (performed in five separate preparations) is shown in Figure 3c. As shown here, prolonged ACh applications at five-minute intervals were followed by a significant, yet incomplete, recovery of network synchrony to approximately 65% of initial Sync Ratio levels. Although absolute Sync Ratio values varied slightly between experiments (see Additional file 2: Figure S2a), all experiments showed a qualitatively similar, partial but significant recovery of network synchrony (as illustrated in detail for one experiment in Additional file 4: Video S1). The time to recovery in these experiments (defined as illustrated in Additional file 5: Figure S4a) ranged from 10 to 16 hours (see Additional file 5: Figure S4b-d). No clear relationships were found between the time to recovery and baseline synchrony or burst characteristics (see Additional file 5: Figure S4b-d).

The initial (approximately 10 hours) periods of suppressed network synchrony were not accompanied by reductions in mean firing rates as compared to pre-ACh applications levels (Figure 3d), indicating that Sync Ratio values can change independently of changes in firing rates (compare Figure 3c and d). On the other hand, synchrony reemergence was associated with some increase in firing rates (Figure 3d). This increase was accompanied by elevated burst rates, (see Additional file 6: Figure S5a), but burst intensity remained smaller than that observed before ACh was applied (see Additional file 6: Figure S5b; see also Additional file 4: Video S1), indicating that the increase in firing rates was probably not entirely attributable to excess bursting.

As mentioned above, on a time-scale of minutes, each ACh application caused a rapid reduction of Sync Ratio values followed by a partial recovery over the next few minutes. These temporal profiles were probably dictated by a combination of network responses to changes in cholinergic input, ACh mixing kinetics, ACh breakdown kinetics, and, possibly, the kinetics of potential adaptive processes. We noted that in the absence of AChE, practically no recovery of the Sync Ratio was observed for >2 hours following a single ACh application (see Additional file 7: Figure S6). Conversely, 10-fold increases in AChE concentrations accelerated Sync Ratio recovery rates only by a factor of <2 (data not shown). The first finding suggests that the short-term (minutes) recovery of the Sync Ratio after each application does not reflect adaptive processes (or spontaneous, non-enzymatic ACh breakdown). The second finding indicates that synchrony recovery rates could be accelerated (albeit modestly) by increasing AChE levels, suggesting that ACh clearance rates played some role in the short term kinetics of synchrony recovery. Interestingly, in some experiments (such as that of Figure 3a) we observed that these short-term recovery kinetics were faster near the end of the experiments; this, however was not clearly evident in all experiments (data not shown). In summary, fixed rates of cholinergic input drive dramatic and sustained network desynchronization, yet over time scales of many hours synchrony gradually reemerges, tending to settle at intermediate, relatively high levels.

### Using feedback to adjust ACh application rates to those needed for sustaining low synchrony levels does not prevent the eventual reemergence of synchrony

The experiments described so far show that the initial suppression of synchrony by moderate cholinergic ‘input’ is followed by its significant recovery over time scales of approximately 20 hours. In these experiments, however, cholinergic ‘input’ was provided at fixed and somewhat arbitrary rates that were indifferent to network synchrony levels (that is, in an ‘open loop’ regime). *In vivo*, feedback plays major roles in the regulation of neuromodulatory outflow [16,72,73], with the cholinergic system, as well as other neuromodulatory systems, encompassed in feedback loops connecting cortical and subcortical regions (findings predicted as early as 1955 [74]). Would the experimental introduction of feedback, serving to adjust cholinergic input rates to instantaneous synchrony levels, allow cholinergic neuromodulation to maintain network synchrony at low levels for very long, perhaps indefinite periods?

To examine this possibility, we constructed a ‘synchrony clamp’ system – a closed-loop controller which estimated synchrony levels in real time and used these estimates to apply ACh at rates required to maintain network synchrony at low predefined levels. In addition, and in analogy to voltage clamp experiments, in which the output current is used to measure changes in membrane conductance [75], the output of this controller allowed us to gauge changes in cholinergic efficacy. Put differently, the controller output, in the form of inter application intervals (IAI, the time intervals between consecutive ACh applications) provided a measure of the degree to which adaptation to cholinergic input had occurred, with increasingly shorter IAIs indicative of increasingly greater adaptation.

The system we used for this purpose was based on the ‘neuronal response clamp’ developed by Marom and colleagues [76-79]. This system was composed of a ‘bare-bones’ personal computer running a real time application (Figure 1a, item 8) which collected data from the 59 MEA channels and calculated the momentary Sync Ratio value (see Methods for further details). A software implementation of a proportional integral (PI) controller compared the calculated Sync Ratio values to predefined, desired Sync Ratio values and used the difference, that is, the error, to adjust the time interval to the next ACh application (as illustrated in Figure 1b).

**Figure 4:**
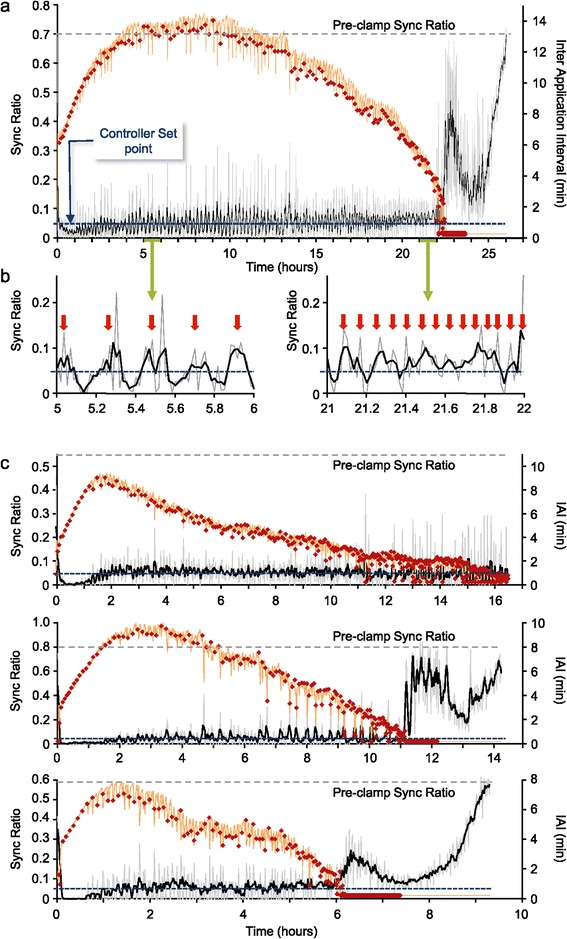
**Using feedback to adjust cholinergic input allows clamping of network synchrony at low levels, but not indefinitely. a)** One closed-loop experiment. Network activity was clamped to a desynchronized activity state (Sync Ratio =0.05). The Sync Ratio, calculated online, is shown as a grey line (corresponding to the left vertical y-axis) and after smoothing with a five-point kernel (black). The inter application interval (IAI), calculated online is shown as an orange line (corresponding to the right vertical y-axis). Each red dot denotes a single ACh application, and the actual time difference from the previous application. The Sync Ratio value preceding the first ACh application is indicated as a dashed gray line. **b)** Enlarged one-hour sections of the plot in a, from early (left) and late (right) stages of the experiment. Red arrows denote application times. **c)** Three additional closed-loop experiments, in which the Sync Ratio was clamped to 0.05. Firing rates for each of the closed-loop experiments are shown in Additional file 8: Figure S7. ACh, acetylcholine.

Experiments were set up as described above for open loop experiments (Figure 1c, phases I-IV). Here, however, instead of applying ACh at fixed intervals, instantaneous IAI values were calculated online once every minute and ACh applications were delivered automatically at these calculated time intervals. Figure 4a,b shows one such experiment in detail, in which the Sync Ratio ‘clamp value’ was set to 0.05, reflecting the fully desynchronized state observed in some open-loop experiments. Activating the closed-loop controller reduced the Sync Ratio from 0.7 to slightly under 0.05 (note that as ACh applications started a few minutes into the experiments, initial Sync Ratio values are observable only at the very beginning of the traces). This led to an initial undershoot of Sync Ratio values which was followed by a gradual adjustment (increase) of IAI values, as shown by the orange line in this figure, until a reasonable clamp of Sync Ratio values was attained after several hours (note that the controller settings were aimed at minimizing fluctuations around the clamp value). During this period, the IAI values required to maintain this low synchrony level hovered around 12 to 13 minutes (Figure 4a). After about 10 hours, however, the IAIs required to sustain this synchrony level started to decrease gradually, ultimately flooring at 15-second intervals (the minimum IAI allowed by the controller). In spite of this 50-fold increase in ACh application rates, synchrony reemerged, escaping the clamp, with Sync Ratio values partially decreasing again only after substantial ACh buildup, caused by the rapid application rates. Qualitatively identical results were obtained in three additional closed-loop experiments (Figure 4c). Closer examination revealed that the time from clamp initiation to eventual collapse back to synchronous activity modes varied from one experiment to the other, ranging from about 6 to about 22 hours, probably reflecting differences in the adaptation rates of different networks as well as other factors related to network to network variability. Here too, no clear relationships were found between the time to collapse (defined as illustrated in Additional file 5: Figure S4e) and baseline network synchrony or burst characteristics (see Additional file 5: Figure Sf-h). Moreover, using IAI values as readouts of adaptation to cholinergic input, we noted that the adaptation process, manifested as decreasing IAI values, could start within a few hours of ACh applications, a fact not immediately apparent in open-loop experiments. Interestingly, firing rates in these experiments remained relatively stable and similar to pre-clamp values until the networks escaped the clamp, during which transient increases in spike rates, associated with volleys of synchronous bursts, were observed (see Additional file 8: Figure S7; Additional file 9: Video S2).

These experiments thus show that even when feedback is used to continually adjust cholinergic input to rates needed for sustaining low desynchronized states, synchrony ultimately reemerges.

### Noradrenergic-induced suppression and reemergence of network synchrony does not occlude subsequent cholinergic-induced synchrony suppression

Conceptually, the capacity to suppress network synchrony in the face of gradual adaptation to neuromodulatory input might be preserved by the activation of other modulatory pathways that exert similar effects but employ different mechanisms. *In vivo*, NA is highly instrumental in promoting brain activity patterns associated with aroused states [20,44]. Does NA also suppress network synchrony in a manner similar to the activities of ACh? Do cortical networks adapt to this neuromodulator as well? If they do, does this adaptation occlude the capacity of cholinergic neuromodulation to suppress network synchrony?

**Figure 5:**
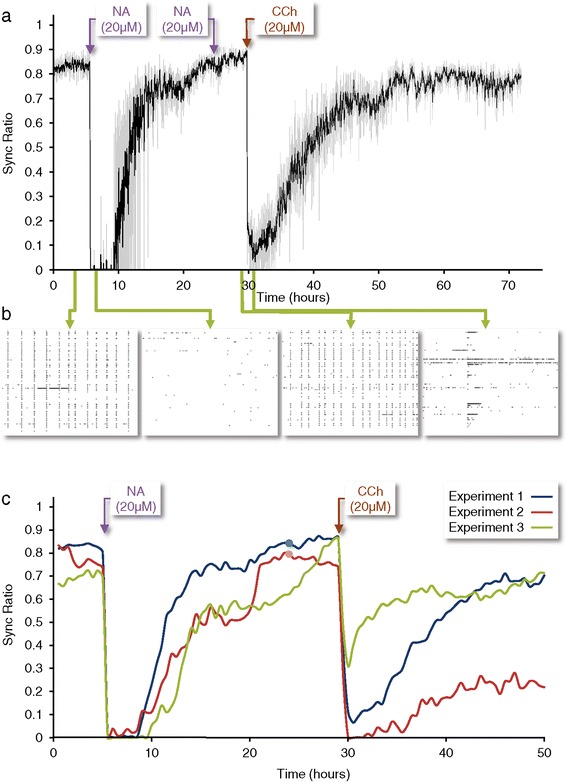
**Network synchrony reemerges following prolonged exposure to NA, but can be re-suppressed by cholinergic input. a)** Evolution of the Sync Ratio in one experiment (grey; same data after smoothing with a five-point kernel is shown in black). After recording baseline activity for >72 hours (of which the last approximately six hours are shown), NA (20 μM) was added directly into the MEA dish and the perfusion media reservoir (see Additional file 11: Figure S8 for similar experiments performed with DA). Nineteen hours later, a second bolus of freshly prepared NA was added to the MEA dish. Twenty-four hours after the first NA addition, CCh (20 μM) was added to the MEA dish and perfusion media reservoir and recording was continued for 40 hours. **b)** Examples of one-minute raster plots from four stages of the experiment as indicated in the figure (green arrows). **c)** Evolution of Sync Ratio (averaged over 30 minute intervals) in three similar experiments (blue line is the same experiment as in a). In two of these, a second bolus of freshly prepared NA was added to the MEA dish 19 hours later (indicated as small circles on blue and red traces). Note the very limited effect of this second NA application. CCh, carbachol; DA, dopamine; MEA, multielectrode array; NA, noradrenaline.

To examine these questions we exposed networks of cortical neurons growing on MEA substrates to NA and followed the immediate and long-term effects of this manipulation on network synchrony. As shown in Figure 5a,b and Additional file 10: Video S3, the addition of NA (20 μM [36,65]) to the MEA dish (and perfusion media) resulted in an immediate reduction in network synchrony which was reflected in drastically reduced Sync Ratio values. Unlike ACh, however, the suppression of network synchrony was also accompanied by an immediate and significant reduction in total firing rates (data not shown), in excellent agreement with a recent *in vivo* study concerning NA, cortical synchrony and firing rates [61]. Reduced Sync Ratio values did not seem to stem from the reduction in firing rates, as this measure is mostly independent of such influences (see above, Additional file 10: Video S3, and another example of such independency below). Interestingly, over the next 10 hours, synchrony (as well as total firing rates) returned to pre-NA application levels. Addition of a second bolus of freshly prepared NA to the MEA dish 19 hours after the first exposure had only minor effects on the Sync Ratio (and on total firing rates), indicating that the recovery of network synchrony was not due to NA breakdown (Figure 5a). The addition of carbachol (CCh; 20 μM) - a non-hydrolyzable analog of ACh - to the same network 24 hours after the first NA application resulted in a dramatic reduction in network synchrony. As before, however, synchrony reemerged over the next 10 to 15 hours (Figure 5a,b; Additional file 10: Video S3).

The evolution of the Sync Ratio in three separate experiments is shown in Figure 5c. The effects of NA and CCh in all experiments were qualitatively very similar, and collectively demonstrate that: 1) NA strongly suppresses network synchrony; 2) prolonged exposure to NA is associated with adaptive or homeostatic processes that restore network synchrony; 3) the capacity of cholinergic neuromodulation to suppress network synchrony is not occluded by prior adaptation to noradrenergic modulation; and 4) synchrony recovers in the presence of both NA and CCh.

We also examined adaptation to a third neuromodulator, dopamine (DA). Unlike ACh and NA, however, relationships between dopaminergic tone, network synchrony and arousal levels are less clear and even somewhat controversial [80-82]. In agreement with this ambiguity, we found, in three separate experiments, that Sync Ratio values were only slightly reduced by single DA applications (500 nM; Additional file 11: Figure S8a; Additional file 12: Video S4). Conversely, firing rates (see Additional file 11: Figure S8b) and burst rates (data not shown) were reduced substantially (see also Additional file 12: Video S4), in agreement with the recent report that DA reduces ‘up’ state prevalence in acute cortical preparations [82]. Interestingly, Sync Ratio values, and most notably, network activity levels, gradually recovered over the course of the next approximately 5 and >10 hours, respectively. These experiments indicate that changes in firing rates induced by various neuromodulators are not necessarily associated with changes in network synchrony and highlight the specific effects of ACh and NA in this respect. They also indicate that prolonged exposure to a third neuromodulator – DA – is also followed by adaptive or reactive processes which occur over many hours, in line with our observations for ACh and NA.

### Periodic ‘withdrawal’ periods restore the capacity of ACh applications to suppress network synchrony in ensuing periods

As mentioned in the introduction, timed cholinergic withdrawal periods (such as those which occur during NREM sleep periods) might ultimately be required to preserve cholinergic efficacy over extended time scales. In the setting of our experimental system, this predicts that withdrawal periods introduced into our experimental protocols will restore the capacity of ACh to suppress network synchrony in subsequent periods.

**Figure 6:**
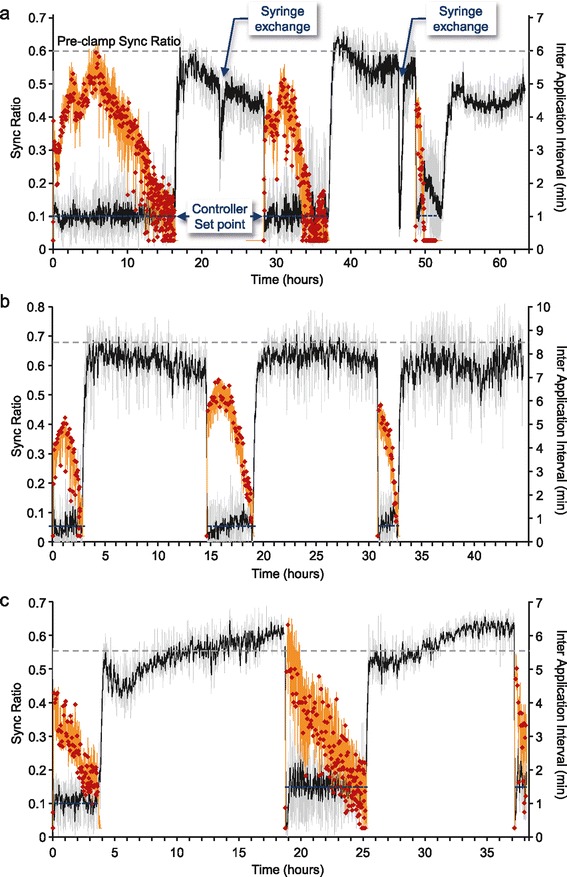
**Withdrawal of cholinergic input for defined periods restores its capacity to suppress network synchrony in subsequent periods.** Three multiple epoch experiments are shown using the same notations as in Figure 4. Network activity was clamped to a desynchronized activity level (Sync Ratio = 0.05 for a, b and 0.1 and 0.15 for c). Following the depletion of the ACh in the application syringe **(a)** or the online calculation of four consecutive IAI values <1.5 minutes **(b,c)**, cholinergic input was removed for 12 hours. After these periods, new closed-loop epochs were initiated, using the same experimental parameters. In the first experiment **(a)**, the syringe was refilled with freshly prepared ACh as indicated in the figure, resulting in some leakage of ACh from the open end of the application needle, and a transient desynchronization of network activity. In the other two experiments **(b,c)** the syringe was not refilled, and the same ACh, prepared at the beginning of the experiment, was used for the entire duration of the experiments. Firing rates for each of the multi-epoch experiments are shown in Additional file 13: Figure S9. ACh, acetylcholine.

To test this prediction, we performed experiments that included three closed-loop epochs, during which network synchrony was ‘clamped’ at low Sync Ratio values, separated by periods during which no ACh was applied (‘withdrawal’ periods). The duration of these periods was chosen to be 12 hours, to mirror the times at which synchrony often started to reemerge following prolonged cholinergic input (Figure 3). As shown in Figure 6a, multiple closed-loop epochs were qualitatively similar to the closed-loop experiments described above. In particular, as in the aforementioned experiments, the demand to maintain synchrony at low predetermined levels was associated with gradual and very significant decreases in IAIs over time. During ‘withdrawal’ periods, synchrony returned almost immediately to the high levels typical of unperturbed networks, as manifested by Sync Ratio values that were similar or even exceeded initial values. Notably, ACh applications delivered after ‘withdrawal’ periods were highly effective in desynchronizing network activity. Moreover, gauging the degree of adaptation from the IAIs needed to clamp network synchrony at the beginning of the second and third closed-loop epochs and comparing these to IAI values at the end of the preceding epochs, indicated that cholinergic efficacy had recovered and that the degree of adaptation to cholinergic input was low.

In the experiment presented in Figure 6a, the ‘withdrawal’ phase of each epoch was started when the ACh application syringe was depleted. Accordingly, the application syringe had to be refilled between epochs, a process that was associated with a small leak of ACh into the culture media (Figure 6a). Moreover, the duration of the closed-loop epochs in this experiment became gradually shorter, reflecting a diminished capability to sustain the network in a desynchronized state. We wondered if this might stem from somewhat extreme demands for low synchrony levels and the consequential, excessive perhaps, cholinergic ‘input’. In subsequent experiments we, therefore, terminated the closed-loop epochs and started the ‘withdrawal’ periods when the IAIs became shorter than 1.5 minutes. This also had the additional benefit of eliminating the need to refill the application syringe between epochs. As shown in Figure 6b,c, the changes in the durations of the closed-loop periods were indeed less systematic. Importantly, however, the general outcome of all experiments was the same: clamping synchrony at low levels was invariably followed by drastic reductions in cholinergic efficacy, whereas cholinergic ‘withdrawal’ was followed by the practically complete recovery of cholinergic efficacy. These experiments also clearly establish that the reduction in cholinergic efficacy was not due to ACh breakdown in the application system, as the same ACh solution was used for the entire duration of these experiments. Interestingly, proactively removing the clamp before escape occurred also precluded the volleys of synchronous bursts such events were associated with (as described above) and resulted in much more moderate changes in firing rates over these long durations (see Additional file 13: Figure S9).

In summary, these experiments demonstrate that withdrawing cholinergic input can restore the capacity of ACh to suppress network synchrony in subsequent periods.

## Discussion

Here we studied adaptation to prolonged neuromodulatory input in networks of cultured cortical neurons. We found that brief ACh applications strongly desynchronized spontaneous network activity, but repeated applications were followed by a gradual recovery of network synchrony. Introducing feedback to adjust cholinergic input levels to waning cholinergic efficacy failed to prevent the reemergence of synchrony even when ACh application rates increased fifty-fold. We then showed that the suppression of network synchrony by a second neuromodulator - NA - was also followed by the gradual reemergence of synchrony. Importantly, this adaptation to continual NA presence did not occlude the subsequent suppression of network synchrony by a cholinergic agonist, yet synchrony ultimately reemerged in the presence of both substances. Finally, we showed that the capacity of cholinergic input to suppress network synchrony could be restored by periodically withdrawing cholinergic input. These findings indicate that prolonged cholinergic (and noradrenergic) input induce slow-acting reactive processes that reduce their efficacy and ultimately negate their capacity to suppress network synchrony.

### Adaptation to prolonged neuromodulation: an inevitable return to network synchrony?

As mentioned in the introduction, neurons and neuronal networks tend to adapt or react homeostatically to changes in input levels or in their milieu [2-6]. Our findings that networks react or adapt to prolonged neuromodulatory input are in line with this well established phenomenon. As neuromodulators affect an enormous number of processes, including attention, arousal, motor function, mood, reward, as well as numerous cellular and molecular processes, adaptive or homeostatic reactions to neuromodulation might be of interest outside the context of network synchrony. Within this limited context, however, the relentless reemergence of synchrony raises interesting questions that touch on the very nature of this widespread phenomenon. It might be asked why network activity consistently falls into this pattern in the absence of neuromodulatory influences, or conversely, falls back into these patterns following prolonged neuromodulatory input.

Starting with the latter, prolonged activation of a particular neuromodulatory pathway might be associated with receptor desensitization, that is, changes in the properties and/or numbers of receptors to that neuromodulator. Thus, for example, it is well established that prolonged exposure to cholinergic agonists is associated with the internalization and, ultimately, down-regulation (degradation and/or reduced synthesis) of muscarinic receptors, which seem to be the major mediators of cholinergic-driven network desynchronization [16,18,57,62] (in particular M1 receptors [65,83]). Where examined, however, internalization was observed to occur rapidly, with membrane receptor levels plateauing within minutes or at most one to three hours following chronic exposure to muscarinic agonists [84-86]. These kinetics seem to be too fast to explain the slow time course of adaptation observed here, a mismatch which also applies to the kinetics of nicotinic receptor desensitization [87]. On the other hand, the slow time course is in better agreement with agonist-driven receptor down-regulation, which has been previously reported to occur over many hours [85]. Yet it also should be noted that this apparently simple explanation is not likely to account for the nearly complete loss of cholinergic efficacy in our experiments. It has been previously shown that even after continual exposure of cultured cortical neurons to CCh for seven days, the amount of M1 muscarinic receptors accessible to an extracellular fluorescent probe was still approximately 50% of nominal levels [88], indicating that a substantial number of receptors were still present even after this long exposure. In line with this report, we previously found that an abrupt pharmacological blockade of cholinergic receptors after nine hours of continuous CCh exposure resulted in an immediate recovery of network synchrony [35], indicating that even after nine hours, receptors were still present and effective.

An alternative, although not mutually exclusive, possibility is that moving networks away from synchronous activity modes is followed by additional reactive processes acting at molecular, cellular and network levels that individually or collectively result in gravitation back toward this mode. Why this gravitation would occur is not entirely clear (see [89] for one substantiated hypothesis) but it does seem to occur also outside the setting of cell culture. Thus, for example, it has recently been shown [90] that in the intact cat brain, suppression of cortical synchrony by thalamic inactivation is followed by cortical synchrony reemergence over time scales remarkably similar to those reported here (12 to 30 hours). In fact, it was concluded that any factor leading to a reduction of synchrony triggers plastic changes that reinstate it. Consequently, and if synchrony represents a strong attractor as prior studies suggest [39,49], escaping it might depend on finely tuned combinations of multiple factors, such as neuronal membrane potential, excitability, probability of neurotransmitter release and synaptic depression. All these (and others) have been previously shown to be affected by (cholinergic) neuromodulation in complex and intricate manners [26,40,55,57,62,65,83,91-95] which differ from one cell type to another [96]. Consequently, the (homeostatic) recovery [25,38] of one or more of these finely tuned factors, in one or more neuronal types, might move the system back into a parameter space within which synchrony naturally emerges [49]. In line with this possibility, we observed that recovery from prolonged neuromodulatory input was often associated with changes in global network activity levels, burst rates and burst intensities (Figure 3, Additional file 6: Figure S5; see also [35,36]). We have previously shown that synchrony reemergence is preceded by excitatory synapse enlargement [35] (see also [97,98]), and thus prolonged neuromodulation seems to drive reactive changes in both neuronal excitability and synaptic properties. Interestingly, it has recently been shown [36] that a neuromodulator cocktail including CCh, NA, serotonin, histamine, dopamine and orexin applied to cortical networks similar to those used here, both suppresses network synchrony and drives widespread changes in gene expression, which do not fully revert even when synchrony spontaneously reemerges. These experimental observations, along with the emergence of synchrony in so many different conditions (prenatal and postnatal development [99,100], NREM sleep, anesthesia), systems (acute, organotypic and cell culture preparations [22-25,27-38,89]) and modeling studies [39,40,48,49] support the possibility that the parameter space for synchrony emergence is quite large, and that avoiding its emergence might be something of a balancing act.

In summary, regardless of the exact nature of the adaptive processes that follow prolonged neuromodulation – changes in receptor properties, receptor abundance, neuronal excitability or network level effects – it seems that the capacity of neuromodulators to suppress synchrony indefinitely might be rather limited.

### Studying adaptation to neuromodulation in cultured cortical networks – confounds and advantages

The reactions to prolonged neuromodulation were studied here in networks of cultured rat cortical neurons exposed to experimental manipulations that do not fully capture many physiological aspects of (prolonged) neuromodulation. While the advantages offered by this model system are substantial (see below), it should also be acknowledged that reduced systems, including the system used here, have certain shortcomings that warrant discussion.

One concern relates to the spatiotemporal structure of cholinergic input used here. Neurons *in vivo* experience cholinergic levels that vary according to the discharge patterns of cholinergic neurons, resulting in both phasic and tonic modes of innervation [101,102]. The phasic mode consists of fast transients of high ACh concentrations, spanning a time scale of seconds and even subseconds [101]. Recent studies suggest that responses to cholinergic innervation can be sensitive to precise (subsecond) timing of cholinergic neuron activity ([91,103] and references therein). In contrast, the regime of cholinergic input in the experiments described here resembled a combination of phasic and tonic modes, with cholinergic concentrations varying more than a hundredfold, but over time scales of many seconds and minutes. Thus, we cannot exclude the possibility that some of our findings were affected by the slow kinetics of cholinergic input and breakdown or by the spatial properties of this input which, if anything, resembled ‘volume’ (extrasynaptic) transmission more than ‘wired’ innervation by cholinergic synapses [101]. In this respect, however, it is worth mentioning that phasic and tonic modes of innervation have been suggested to co-exist and play complementary roles, for example in fostering cue detection and general alertness [102]. Moreover, considering that basal forebrain cholinergic neurons tend to discharge at approximately 7 Hz rhythmic bursts [104], that brief stimulation of these neurons induces cortical desynchronizations that persist for up to 20 seconds [57], and that the kinetics of neuronal muscarinic ACh receptors are rather slow [105], changes in cholinergic tone occurring over many second time scales are probably of significant physiological importance.

A second issue concerns the time course of adaptation to cholinergic input and the duration of ‘withdrawal’ periods described here. We found that the general time scale of synchrony reemergence in our experiments (many hours) was comparable with the time scales of circadian synchrony modulation *in vivo*. However, beyond this general similarity, the actual time scales should not be taken too literally; beyond the obvious fact that these were measured in a reduced system, the level of cholinergic input the neuronal networks were exposed to here was somewhat arbitrary, and it is not clear how these relate to cumulative cholinergic exposure or its fine temporal structure *in vivo*. Along the same line, in our experiments, cholinergic ‘withdrawal’ periods were chosen to be 12 hours. The introduction of these withdrawal periods was inspired by the low neuromodulatory levels occurring during NREM sleep periods. Physiologically, however, NREM periods are separated by short REM periods characterized by especially high cholinergic tone and desynchronized cortical activity [18,106]. In humans, each cycle of NREM and REM sleep lasts approximately 1.5 hours, suggesting that the restoration of efficacy, at least in the case of cholinergic innervation, might occur over shorter time scales. Indeed, in three experiments in which a single ACh application was administered approximately two hours after the end of a closed-loop epoch, effective desynchronization was observed (data not shown). Yet there were also indications that recovery from adaptation might occur over multiple time scales – for example, in the experiment shown in Figure 6a, closed-loop epochs tended to become progressively shorter, which might be taken to indicate that a slow component of the adaptation process persists for longer periods (akin, perhaps, to the ‘sleep debt’ that follows prolonged sleep deprivation).

While the reduced system used here has shortcomings, it also has important advantages in the context of the questions addressed here. Specifically, the experiments described above require full control over the inputs of the preparations to rule out the possibility that the reemergence of synchrony was imposed by external sources. Thus, for example, it is conceptually difficult to imagine how one could perform the same experiments *in vivo*, isolating the monitored networks from the influences of external excitatory and inhibitory sources, circadian systems and additional neuromodulators. Even if one could selectively stimulate cholinergic [50] or noradrenergic [51] neurons for prolonged periods, it would be impossible to unequivocally attribute the reemergence of synchrony to adaptation to these neuromodulatory substances and rule out the possibility that these activity patterns, which typically coincide with inattentive or sleep states [13,107], were imposed by other systems.

Thus, at present, the reduced system used here provides unique opportunities to examine these questions in an isolated, well-controlled fashion, with good access to network activity properties and the possibility to use advanced closed-loop techniques to expose and record adaptation kinetics. Interestingly, a very recent *in vivo* study further supports the utility of this reduced system to study these questions. In this study [61] it was shown that whereas both the cholinergic agonist CCh and NA abolish synchronous slow oscillations in the intact rat barrel cortex, the effects on spontaneous firing rates differed: as in our own findings, CCh had practically no effect on firing rates whereas NA significantly suppressed these. It thus seems that in spite of differences in network organization, neuronal makeup and maturity, the relationships between network activity characteristics and neuromodulation are largely preserved in this reduced system.

## Conclusions

What might be the general implications of our findings? Limitations of the system used here notwithstanding, the findings seem to tie together four themes, namely synchrony, neuromodulation, homeostasis and sleep, in a manner that is rather unconventional yet potentially important. As mentioned in the introduction and in the discussion above, the widespread occurrence of synchrony in many conditions and systems would seem to suggest that it constitutes a ‘default’ activity mode and that some active process might be required to move networks out of it. In fact, as early as 1949, Hebb [108] postulated that this form of synchrony, which ‘appears at birth in the large slow waves of the infant’s electroencephalogram and in the sleep or coma of older subjects’ represents an ‘intrinsic organization of cortical activity’, which needs to be ‘interrupted’ as a ‘precondition of the normal adult waking behavior’ (a conclusion echoed in recent studies documenting tight temporal relationships between synchrony emergence and loss of consciousness [66,67]). It has been suggested that input from external sources serves in this function and, thus, hypersynchrony in reduced systems (such as cell culture) might reflect the lack of such input [70]. Indeed, previous studies have shown that random stimulation patterns whose intensity is continuously adjusted in a closed loop manner can transform synchronous activity into more asynchronous firing patterns [70] and that this strategy can maintain such patterns for six hours [109]. However, in more physiological settings, sensory input *per se* does not always seem to be sufficient (for example, [110,111]). On the other hand, the indispensable role of central neuromodulatory systems in ‘interrupting’ these forms of synchrony has been firmly established by numerous studies [44,50,51,53-60,112], beginning with the pioneering work of Morruzi and Magoun [52]. Given the findings described here, as well as the well-established tendency of neurons and neuronal networks to react or adapt to prolonged perturbations, it might be expected that the efficacy of neuromodulator-based ‘interruptions’ might diminish with time. In line with this expectation, a progressive reemergence of synchrony has been reported in sleep-deprived but behaviorally awake rats which continue to interact with their environments [13,107] (see also [113]).

In spite of the many intriguing parallels between neuromodulation, cortical synchrony, sleep and arousal on the one hand, and the findings described here, on the other, the applicability of our findings to the intact organism remains speculative. Yet, the many similarities with the underlying biophysics of network synchrony *in vivo*, as well as the similar initial reactions to the neuromodulators studied here indicate that the long-term reactions to these neuromodulators and the reemergence of synchrony these entail might also apply to the intact brain. If this parallelism is extended, our findings might also imply that even with the additional degrees of freedom provided by multiple, partially redundant neuromodulatory systems [18], ‘withdrawal’ periods might ultimately be essential for restoring neuromodulator efficacy and, in particular, their ability to counter the tendencies of synchrony to emerge. We hypothesize that this withdrawal is best achieved during tightly controlled sleep periods, which are thus viewed as an unavoidable price of prolonged neuromodulator-driven arousal.

## Methods

### Primary cultures of dissociated cortical neurons

Primary cultures of rat cortical neurons were prepared as described previously [69] using a protocol approved by the Technion committee for the supervision of animal experiments. Briefly, cortices of one- to two-day-old Sprague-Dawley rats were dissected, dissociated by trypsin treatment followed by trituration using a siliconized Pasteur pipette. A total of 1 to 1.5*10^6^ cells were then plated on thin glass MEA dishes (MultiChannelSystems (MCS), Reutlingen, Germany) whose surface had been pre-treated with polyethylenimine (Sigma, St. Louis, MO, USA) to facilitate cell adherence. Cells were initially grown in media containing minimal essential medium (MEM, Sigma), 25 mg/L insulin (Sigma), 20 mM glucose (Sigma), 2 mM L-glutamine (Sigma), 5 μg/ml gentamycin sulfate (Sigma) and 10% NuSerum (Becton Dickinson Labware, Bedford, MA, USA). The preparation was then transferred to a humidified tissue culture incubator and maintained at 37°C in a gas mixture of 5% CO_2_, 95% air. Half the volume of the culture medium was replaced four times a week with feeding media similar to the media described above but devoid of NuSerum, containing a lower L-glutamine concentration (0.5 mM) and 2% B-27 supplement (Invitrogen, San Diego, CA, USA).

### Electrophysiological recordings

The thin glass MEA dishes used here contained 59, 30 μm diameter electrodes arranged in an 8 x 8 array, spaced 200 μm apart. The flat, round electrodes are made of titanium nitride, whereas the tracks and contact pads are made of transparent indium tin oxide. A submerged platinum wire loop connected to a custom designed cap covering the MEA dish (see below) was used as a common reference.

Electrophysiological extracellular recordings from MEA dishes were performed using a commercial 60-channel headstage (inverted MEA-1060-BC, MCS) with a gain of 53x and frequency limits of 0.02 to 8,500 Hz. This signal was further filtered with frequency limits of 150 to 3,000 Hz and amplified (20x) using a filter/amplifier (FA60S-BC, MCS), and then digitized (PD2-MF-64-3 M/12H, UEI, Walpole, MA, USA) at 16 KSamples/second per channel. All data were stored as threshold crossing events with the threshold determined according to one to two seconds of pre-experiment analog voltage recordings, and calculated separately per channel as (mean - 9 x STD) of the voltage trace. Data were then analyzed offline (in open-loop experiments) using custom written scripts in Matlab (MathWorks, Natick, MA, USA) or online (in closed-loop experiments) as explained below.

Combined intracellular whole-cell patch clamp and extracellular recordings from the MEA dish were performed by modifying a MEA recording setup mounted on an inverted microscope to allow for access to the network with a micromanipulator. Additionally, an intracellular recording amplifier (Axoclamp-700B, Molecular Devices, Sunnyvale, CA, USA) was added, from which all data were streamed to a free channel of the MEA recording system, guaranteeing perfect synchronization of extracellular and intracellular recordings. In some combined recordings experiments the culture medium of cortical preparations growing on MEA dishes at 25 to 29 Days in vitro (DIV) was gently replaced by Tyrode’s physiological solution (NaCl 119 mM, KCl 2.5 mM, MgCl_2_ 2 mM, HEPES 25 mM, CaCl_2_ 2 mM and glucose 30 mM, pH 7.4) whereas in others, the original media was not replaced. Large, presumably-pyramidal cells were visualized with brightfield optics using a 40×, 1.3 N.A. Fluar objective. Whole-cell patch-clamp recordings were made at 28°C from the cells soma (n = 6 neurons, 3 networks). Recording pipettes (4 to 9 MΩ) were pulled from filament-containing borosilicate glass capillaries (World Precision Instruments, (WPI) Sarasota, FL, USA) and filled with a solution at pH 7.2 containing: K-gluconate 115 mM, KCl 20 mM, HEPES 10 mM, Mg-ATP 2 mM, Na_2_-ATP 2 mM, GTP 0.3 mM and Na_2_-phosphocreatine 10 mM [114]. Intracellular voltage recordings (current-clamp mode) were pre-amplified (10x), bridge-balanced and then further amplified (5x) and low-pass filtered at 10 KHz with MCP-Plus variable gain filter amplifiers (Alpha Omega, Nazareth, Israel). Extracellular recordings were performed using a commercial 60-channel headstage/amplifier (Inverted MEA1060, MCS) with a gain of 1,024x and frequency limits of 1 to 5,000 Hz. The headstage/amplifier was connected to a bank of 64 variable gain filter amplifiers (MCP-Plus, Alpha Omega), with frequency limits set to 400 to 4,000 Hz, and the gain set to 10x. Data were digitized (16 bit, +/- 5 V voltage range) by two parallel 5200a/526 A/D boards (Microstar Laboratories, Bellevue, WA, USA) at 12/24 KSamples/second (extracellular/intracellular) per channel. Data acquisition was performed using AlphaMap (Alpha-Omega, Nazareth, Israel) and exported to MATLAB for analysis. For extracellular recordings, threshold crossing events were detected offline with the threshold set individually per channel to (mean - 7 x STD) of the voltage trace.

### Long-term MEA recordings

MEA dishes were covered with a custom designed cap containing inlet and outlet ports for perfusion media and air mixtures, a reference ground electrode and a removable rubber connector used to stabilize the microinjection application needle (see below). The MEA dish was continuously perfused with feeding media (described above) at a rate of 2.5 ml/day by means of a custom built perfusion system based on an ultra-slow flow peristaltic pump (Instech Laboratories Inc., Plymouth Meeting, PA, USA) using an imbalanced set of silicone tubes. The tubes were connected to the dish through the appropriate ports in the custom designed cap. A sterile mixture of 95% air/5% CO_2_ was continuously streamed into the dish at very low rates through a third port with flow rates regulated by a high precision flow meter (Gilmont Instruments, Barrington, IL, USA). The base of the headstage/amplifier was heated to 37°C using resistive elements, a temperature sensor and a controller.

### Pharmacological manipulations

ACh and AChE (Sigma-Aldrich, Rehovot, Israel) were applied to the culture media using a custom built application system (see section below). AChE (from *Electrophorus electricus*, electric eel) was added both to the MEA dish and to the perfusion media to a final concentration of 0.1 U/ml, while ACh was added only to the MEA dish. The ACh concentration in the application syringe was 20 mM, with each application of 1 μl diluted by a factor of approximately 1:2000, resulting in a calculated final concentration of 10 μM in the dish (ignoring breakdown by AChE). Calculations based on manufacturer-provided data on enzyme activity suggest that 0.1 U/ml AChE can hydrolyze a single application of 10 μM ACh within approximately 5.7 seconds, yet this was obviously a coarse estimation, not taking into account our specific experimental conditions.

NA,CCh and DA (Sigma-Aldrich) were each applied to the culture media by diluting them into 100 μL of medium drawn from the MEA dish. The mixture was subsequently returned to the dish and mixed gently. Applications to the dish were complemented by simultaneous addition to the perfusion media to a final concentration of 20 μM (NA and CCh) and 500 nM (DA).

### Application and mixing system

ACh applications were performed using an automated application system composed of a syringe (500 μl, Hamilton, Bonaduz, Switzerland, model 750 for ACh) mounted on a high-precision pump (UltraMicroPump III, WPI) controlled by an automated controller (Micro4, WPI). The syringe was connected to the MEA dish by a narrow tube, at the end of which was a needle, inserted and stabilized by a rubber connector placed within the custom built cap of the MEA. The open end of this needle was immersed in the culture media, hovering about 1 mm above the neuronal network.

A continuous mixing system was used to facilitate the spread of ACh in the culture media and its subsequent breakdown by AChE. This system was constructed as a flywheel-crankshaft system using a small electric motor and a metal strip, which was connected to the piston of a 1 ml syringe (Hamilton, model 1001). This syringe was connected by silicon tubing to a narrow glass tube immersed within the culture media and facing the MEA dish sidewalls, so that the media flow would be directed away from the neuronal networks. After many preliminary experiments to determine the effects of the system on baseline network activity and neuronal vitality, we settled on mixing volumes of 200 μl at rates of 10 volumes/minute. Control experiments using a concentrated dye (sulfoRhodamine B, 330 uM) revealed that these rates resulted in dye dispersal within 60 to 120 seconds (data not shown).

### Experimental procedure

Each open- or closed-loop experiment was preceded by the following preparatory phases: after at least 24 hours of slow perfusion (Figure 1c, Phase I), the continuous mixing system was activated (Figure 1c, Phase II). The activation of the mixing system was followed by some additional inter-burst firing recorded from a few electrodes, the extent of which varied somewhat among different preparations, resulting, sometimes, in slight decreases in Sync Ratio values (see Additional file 1: Figure S1, second raster from top, for an example of a preparation in which these effects were most apparent). Twelve to twenty-four hours after activating the mixing system, AChE (0.1 U/ml) was added directly to the culture medium in the MEA dish and to the perfusion medium reservoir and activity was recorded for at least 30 minutes (Figure 1c, Phase III). AChE addition had no apparent effect on network activity or synchrony levels (Additional file 1: Figure S1, third raster from top). At this point, the syringe containing the ACh solution was mounted on the syringe pump and the application needle was inserted into the MEA dish (Figure 1c, Phase IV). Following these phases, experiments were started by initiating automated ACh applications in either an open- or closed-loop regime (Figure 1c, phase V).

### Real-time system

Open- and closed-loop experiments were performed using a real-time system (although only closed-loop experiments required the real-time component). The real-time system was composed of a bare-bones personal computer running a real time application over Simulink’s xPC target (Mathworks). The application was composed of modular units designed to perform different parts of the experimental protocol in real time. These included the detection of threshold crossing events, the quantification of network synchrony, an implementation of a PI controller (in closed-loop experiments, see below) and the execution of ACh applications through commands issued to the microapplication system at predefined/calculated (open/closed-loop) times. In addition, this computer periodically streamed the recorded data, as well as information on network synchrony and ACh application times, to a second computer (running Mathworks Matlab and Microsoft Windows) which served as a host for user interface and data storage.

### Feedback loop and the PI controller

Closed-loop experiments were executed by a Simulink implementation of a feedback loop in the real-time computer. This loop included the following steps:

1. Network activity was continuously sampled from the 60 electrodes.
2. Once a minute, a single value of the network synchrony measure, Sync Ratio (see below), was calculated and subtracted from a predetermined set point, the clamp value, to produce the error (Eq. 1).
3. The error was used by the PI controller (Eq. 2) to calculate the IAI.
4. The IAI value was added to the previous application time to produce the next ACh application time. The Sync Ratio, error value and IAI were updated every one minute, resulting in the online tuning of the next application time: if the calculated IAI value increased, the next application time was delayed to reflect the new IAI value. Conversely, if the IAI value decreased, the next application time was moved backwards (if this resulted in an application time that had already elapsed, the application was performed immediately).
5. When the application time was reached, a command was sent to the application system controller, and the ACh syringe applied 1 μl to the dish.

1 $$ {e}_n= Sync\; Rati{o}^{*}- Sync\; Rati{o}_n $$

In single-epoch closed-loop experiments, this loop was executed until the ACh application syringe was depleted. In multi-epoch experiments, a 12 hour ‘withdrawal’ period commenced when the ACh application syringe was depleted (first experiment, Figure 6a) or when the IAI value was <1.5 minute in four consecutive minutes (two additional experiments, Figure 6b,c). After this ‘withdrawal’ period, a new closed-loop phase was started, then another ‘withdrawal’ and so on.

The PI controller was realized using a Simulink implementation. As mentioned above, the input to the controller was the error signal (*e*_*n*_), which was used to calculate IAI_n_ according to the following equation:

2 $$ IA{I}_n=\left(IA{I}_{Baseline}+{g}_P\cdot {e}_n+{g}_I\cdot {\displaystyle {\sum}_{i=1}^n{e}_i}\right)*{g}_{convert} $$

where g_P_, g_I_ are the proportional and integral gain parameters which were set to 1 and 0.2, respectively, in all experiments. g_convert_ is simply a conversion gain into relevant units of IAI (minutes) for the sake of convenience. IAI_baseline_ is the baseline interval between ACh application, which was set to different values (ranging from 5 to 8.5 minutes) in different experiments, determined arbitrarily or according to a few test applications performed prior to the closed-loop phase in some experiments. This value affected only the beginning of the closed-loop phase (indeed, we found an undershoot of the Sync Ratio in all experiments), as the integral component of the controller corrects for this initial bias over time.

### Data analysis - quantifying network synchrony

To quantify the effects of neuromodulation over time, an accurate and reliable measure of network synchrony was needed. Although we and others [36] have previously used the Burstiness Index (BI) developed by Wagenaar and co-workers [70] for this purpose, in the course of preliminary experiments we found that this measure can misrepresent the degree of network synchrony under some circumstances. For example, reductions in overall network activity levels during periods of clearly asynchronous activity, often lead to spuriously higher BI values. Conversely, significant increases in burst rates, which often followed prolonged elevations of cholinergic tone, were often associated with reduced BI values. We, therefore, devised a different measure, which we termed the Sync Ratio. The Sync Ratio measure robustly distinguishes between synchronous and asynchronous activity modes (see Additional file 3: Figure S3) and was, therefore, used in all experiments. The premise of this measure is that synchrony, by definition, implies that many neurons across the network are approximately simultaneously active. Therefore, the measure is based on the number of active electrodes within a short (10 mseconds) time bin, instead of focusing on the temporal structure of action potentials recorded from single electrodes.

The Sync Ratio measure was calculated for one minute (or 0.5 hour in Figure 5c and Additional file 3: Figure S3d, and ten minute in Additional file 8: Figure S7, Additional file 11: Figure S8 and Additional file 13: Figure S9) time intervals by: 1) counting the number of active electrodes (that is, the number of electrodes from which an action potential was recorded at least once within that bin) in consecutive 10 msecond bins; 2) summing the active electrode counts in bins in which this count exceeded a threshold (above which activity was considered to be synchronous); and 3) dividing this sum by the sum of all active electrode counts in the one minute interval, to give the Sync Ratio:

3 $$ Sync\; Ratio=\frac{{\displaystyle \sum \left( active\kern0.24em  electode\kern0.24em  counts>th\right)}}{{\displaystyle \sum \left( Total\kern0.24em  active\kern0.24em  electode\kern0.24em  counts\right)}} $$

The threshold for the Sync Ratio was defined as the number of electrodes per bin for which the probability of exceeding it by chance was <0.0001 had activity in the network occurred randomly and independently at each electrode. This was calculated by: (1) calculating the average spike rate per electrode during a half hour interval prior to ACh applications; (2) using the average spike rates to create a surrogate data spike train for each electrode according to a Poisson distribution; (3) calculating the active electrode per bin (10 mseconds) counts for the surrogate data; (4) determining the threshold for which the Sync Ratio would be <0.0001 for the surrogate data.

Burst detection was performed for one minute time intervals (as previously described [33]) with the following changes: detection was defined when activity crossed a threshold of N number of action potentials recorded throughout the electrode array within a T millisecond time bin, with N and T defined as 40% of active electrodes and 10 mseconds, respectively. Moreover, the burst starting point and termination were defined when activity crossed 10% of the threshold defined above, and a refractory period of 80 mseconds was applied to burst detection analysis (no burst detection for 80 mseconds following previous detection). Burst rates and burst intensity were averaged over one minute time intervals, defined as the number of bursts per second and the average number of action potentials within a burst, respectively.

All data were exported to Matlab and analyzed using custom written scripts. Final graphs were prepared using Microsoft Excel. All final figures were prepared using Microsoft PowerPoint.

## Additional files

Additional file 1: Figure S1. Changes in network activity characteristics during preparatory phases. Example of network activity characteristics during each of the preparatory phases described in Figure 1c, illustrated by representative one-minute raster plots of activity recorded from all electrodes in this particular network, and the Sync Ratio values obtained during these periods. In most experiments, these preparatory phases had only minimal effects on network synchrony, but in some experiments (as in the one shown here), activation of the mixing system (Phase II) induced some asynchronous firing, resulting in some reduction of Sync Ratio values. Consequently, Sync Ratio values measured during this phase were considered to represent pre-ACh application Sync Ratio values. Additional file 2: Figure S2. Baseline network activity characteristics are stable. **a)** Sync Ratio, **b)** burst rate and **c)** burst intensity measured for the five networks of Figure 3 during 12 hour periods at the beginning of these experiments (Phase I in Figure 1c). Note the relative stability of these measures in all networks. See Methods for further details on burst detection. Additional file 3: Figure S3. The Sync Ratio as a measure of network synchrony. The premise of this measure is that synchrony, by definition, implies that many neurons across the network are almost simultaneously active. Therefore, the measure is based on the number of electrodes concomitantly active within a small time window. **a)** One-minute raster plots of network activity recordings in normal medium (left) and carbachol (CCh; right) showing distinctly different activity patterns. In normal media, activity in all electrodes is highly synchronous, whereas in CCh, activity is much more tonic and asynchronous. Time is given in minutes from the beginning of recording. **b)** Enlarged portions (4.8 seconds) from each trace in a. **c)** Active electrode per bin counts (bin =10 mseconds) over 4.8 seconds from each trace shown in b. The dashed line depicts the threshold defined for the Sync Ratio calculation (th =7), clearly discriminating between synchronous events and desynchronized firing. Sync Ratio values denoted were calculated over the 4.8 second data traces. The inset in the left panel shows two synchronous events at a higher magnification, with suprathreshold bins highlighted in red. **d)** Network synchrony estimated by two measures - Burstiness Index (BI, calculated according to Wagenaar and colleagues [70], with some adjustments of parameters as previously described [35]) and Sync Ratio, calculated at half-hour intervals for previously published experiments [35] in which CCh was used to elevate cholinergic tone. Both measures show the same trends; however, the Sync Ratio, as opposed to the BI, is less affected by increased burst rates near the end of the experiment and fully recovers to pre-CCh levels. Additional file 4: Video S1. Effects of ACh applications on network activity in one open-loop experiment. In this video, each frame shows a one-minute raster plot of activity measured from the MEA 59 electrodes. Each dot represents an action potential. ACh was applied at five minute intervals. The first five minutes preceding the first ACh application are displayed slowly to provide a better impression of basal network synchrony. Activity between hours 3 and 10 (180 to 600 minutes) was edited out to keep the file size within reasonable limits. Note the dramatic change in activity patterns induced by ACh, and the gradual reappearance of synchrony as manifested by vertical ‘lines’ in the raster plots. Additional file 5: Figure S4. Synchrony reemergence time is mostly independent of network baseline activity characteristics. **a)** Illustration of the procedure used to determine the Time to Recovery in the open loop experiments of Figure 3. To that end, Sync ratio values determined at one-minute intervals were smoothed with a five point kernel and the time at which the Sync Ratio exceeded the half recovery point, defined as ≡ *min* _ *val* +0.5 ⋅ (*max* _ *val* ‐ *min* _ *val*) was obtained. No obvious relationships were detected between the Time to Recovery and **b)** burst rates; **c)** burst intensities; and **d)** Sync Ratio values measured during 12 hour baseline recording periods (phase I in Figure 1c) in all open loop experiments of Figure 3. **e)** Illustration of the procedure used to determine the Time to Collapse in the closed-loop experiments of Figure 4. To that end, the time, in each experiment, at which the inter application interval (IAI) first reached 0.25 minutes was obtained. No obvious relationships were observed between the Time to Collapse and **f)** burst rates; **g)** burst intensities; and **h)** Sync Ratio values measured during 12 hour baseline recording periods (phase I in Figure 1c) in all closed-loop experiments of Figure 4. AP, Action Potentials. Additional file 6: Figure S5. Changes in burst rates and burst intensities following prolonged ACh applications. **a)** For each open loop experiment of Figure 3, burst rate values, averaged over 30 minutes, were normalized to the average burst rates measured during 12 hour baseline recording periods (phase I in Figure 1c). Left – baseline periods. Right – open loop ACh application periods (phase V in Figure 1c). **b)** A similar analysis for burst duration. Averages ± SEM for five experiments. See Methods for further details on burst detection. Additional file 7: Figure S6. The presence of AChE is required for phasic-like cholinergic input. The Sync Ratio is depicted over time following a single application of ACh at t =0 with no AChE applied (red) or with AChE in the media (blue; average of two repeats. This AChE concentration (0.1 U/ml) was used in all experiments. Additional file 8: Figure S7. Comparisons of firing rates and Sync Ratio values in closed-loop experiments. Sync Ratio (blue) and firing rate (red) values, both averaged over 10 minute bins, are shown for each closed-loop experiment shown in Figure 4. In all experiments Sync Ratio values were significantly lower than pre-clamp values, while firing rates were not significantly altered or changed only slowly after the initiation of the clamp (blue and red dashed lines, accordingly). The escape from the clamp was associated with temporary increases in firing rates due to transient periods of volleys of synchronous bursts that were associated with this escape after which firing rates tended to subside while synchrony gradually approached pre-clamp levels (see also Additional file 9: Video S2). AP, Action Potentials. Additional file 9: Video S2. Effects of ACh applications on network activity in one closed-loop experiment. Each frame is an activity raster plot as explained in Additional file 4: Video S1. The first minute preceding the first ACh application is displayed slowly to provide a better impression of basal network synchrony. Activity between hours 3.5 and 8 (210 to 480 minutes) was edited out to keep the file size within reasonable limits. Note the sudden emergence of synchrony near the end of the experiments as the network escaped the clamp. Same experiment as in Figure 4c (middle panel) and Additional file 8: Figure S7 (third panel from top). Additional file 10: Video S3. Effects of NA and CCh on network activity in one experiment. Each frame is an activity raster plot as explained in Additional file 4: Video S1. Activity between hours 13.2 and 22.0, 31.7 and 38, and 40.8 and 47.6 (789 to 1,322; 1,905 to 2,282; 2,449 to 2,856 minutes) was edited out. Same experiment as shown in Figure 5a, and c (blue line). Additional file 11: Figure S8. Dopamine effects on network synchrony and firing rates in networks of cultured cortical neurons. After recording baseline activity for at least 72 hours (of which the last 25 hours are shown), DA (500nM) was added directly into the MEA dishes and the perfusion media reservoirs. The figures show the evolution of the Sync Ratio **(a)** and normalized firing rates **(b)** in these experiments, averaged over 10 minute periods. Note the very limited effect of DA on the Sync Ratio, and the more pronounced effect on firing rates. Note also that the Sync Ratio recovered to pre-application levels faster than firing rates did. Averages ± SEM for three separate experiments. AP, Action Potentials. Additional file 12: Video S4. Effects of DA on network activity in one experiment. Each frame is an activity raster plot as explained in Additional file 4: Video S1. Activity between hours 26.5 and 31.6 (1,590 to 1,898 minutes) was edited out. Note that DA had no clear effects on network synchrony (compare with Additional files 4, 9 and 10: Videos S1-S3). Additional file 13: Figure S9. Firing rates and the Sync Ratio in multi-epoch experiments. Sync Ratio (blue) and firing rate (red) values, both averaged over 10 minute bins, are shown for each multi-epoch experiment shown in Figure 6. Periods in which the synchrony clamp was in effect are shown as thick orange lines. Note that firing rates and Sync Ratio values do not co-vary consistently. AP, Action Potentials.

## Abbreviations

ACh: acetylcholine
AChE: acetylcholine esterase
CCh: carbachol
DA: dopamine
EEG: electroencephalogram
IAI: inter application intervals
MEA: multielectrode array
NA: noradrenaline
NREM: non rapid eye movement
PI: proportional integral
