# Supplementary figures and images for: Adaptation to prolonged neuromodulation in cortical cultures: an invariable return to network synchrony
Source: BMC Biol. 2014 Oct 23;12:83. doi: 10.1186/s12915-014-0083-3 (PMC4237737; doi:10.1186/s12915-014-0083-3)

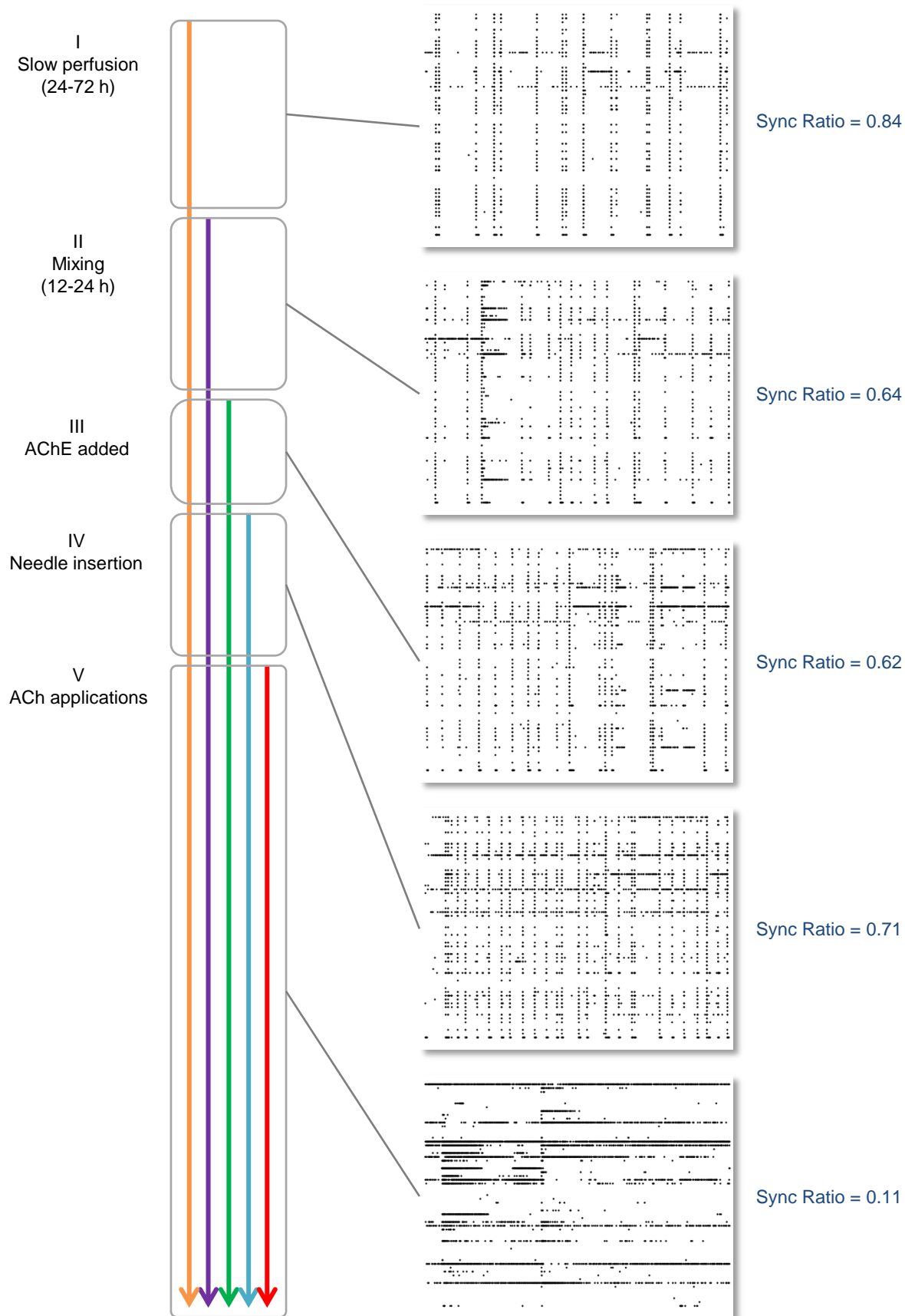

Kaufman et al.  
Supplementary Figure 1

Supplement: Additional file 1: Figure S1. — Changes in network activity characteristics during preparatory phases. Example of network activity characteristics during each of the preparatory phases described in Figure 1c, illustrated by representative one-minute raster plots of activity recorded from all electrodes in this particular network, and the Sync Ratio values obtained during these periods. In most experiments, these preparatory phases had only minimal effects on network synchrony, but in some experiments (as in the one shown here), activation of the mixing system (Phase II) induced some asynchronous firing, resulting in some reduction of Sync Ratio values. Consequently, Sync Ratio values measured during this phase were considered to represent pre-ACh application Sync Ratio values. [file 12915_2014_83_MOESM1_ESM.pdf]

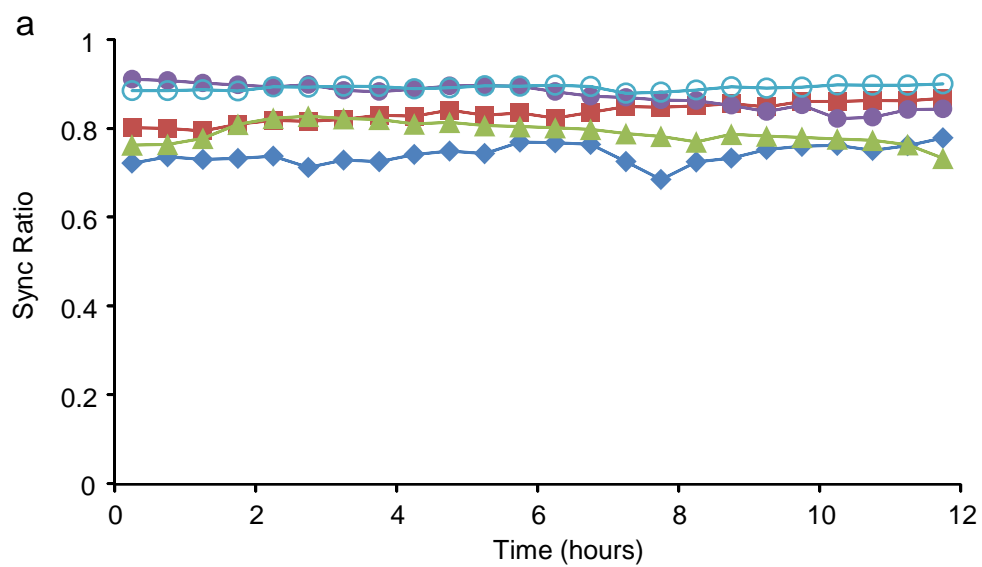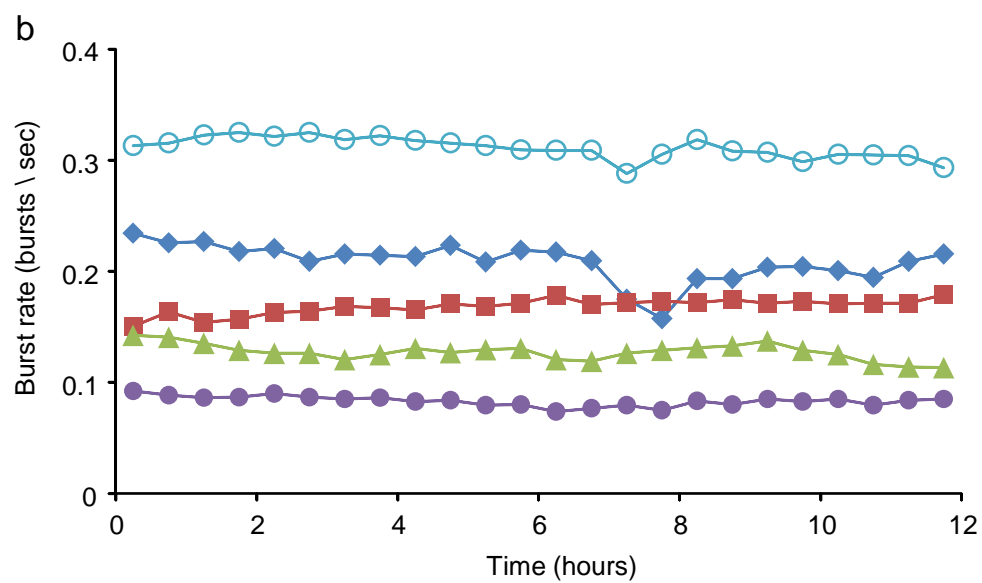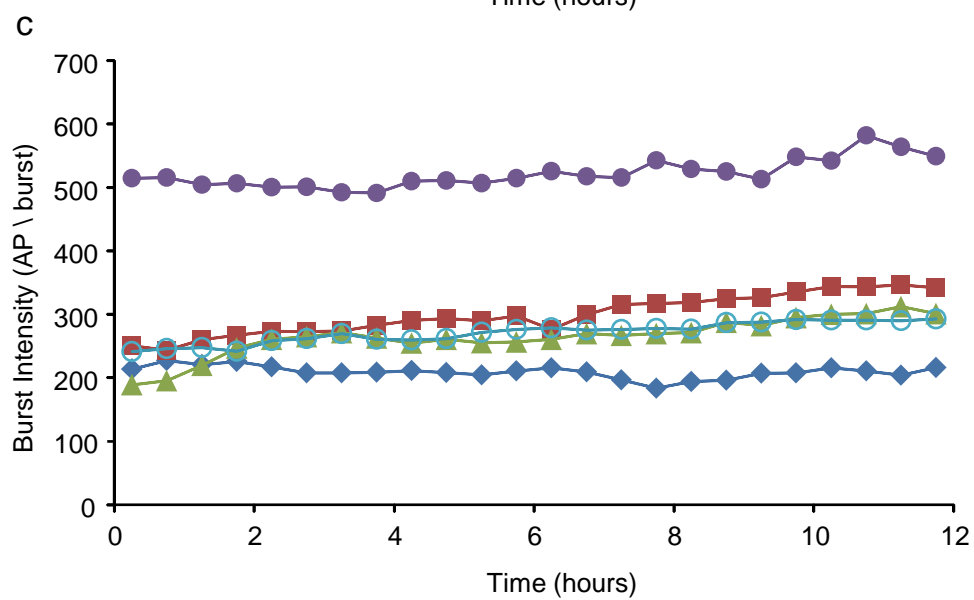

Supplement: Additional file 2: Figure S2. — Baseline network activity characteristics are stable. a) Sync Ratio, b) burst rate and c) burst intensity measured for the five networks of Figure 3 during 12 hour periods at the beginning of these experiments (Phase I in Figure 1c). Note the relative stability of these measures in all networks. See Methods for further details on burst detection. [file 12915_2014_83_MOESM2_ESM.pdf]

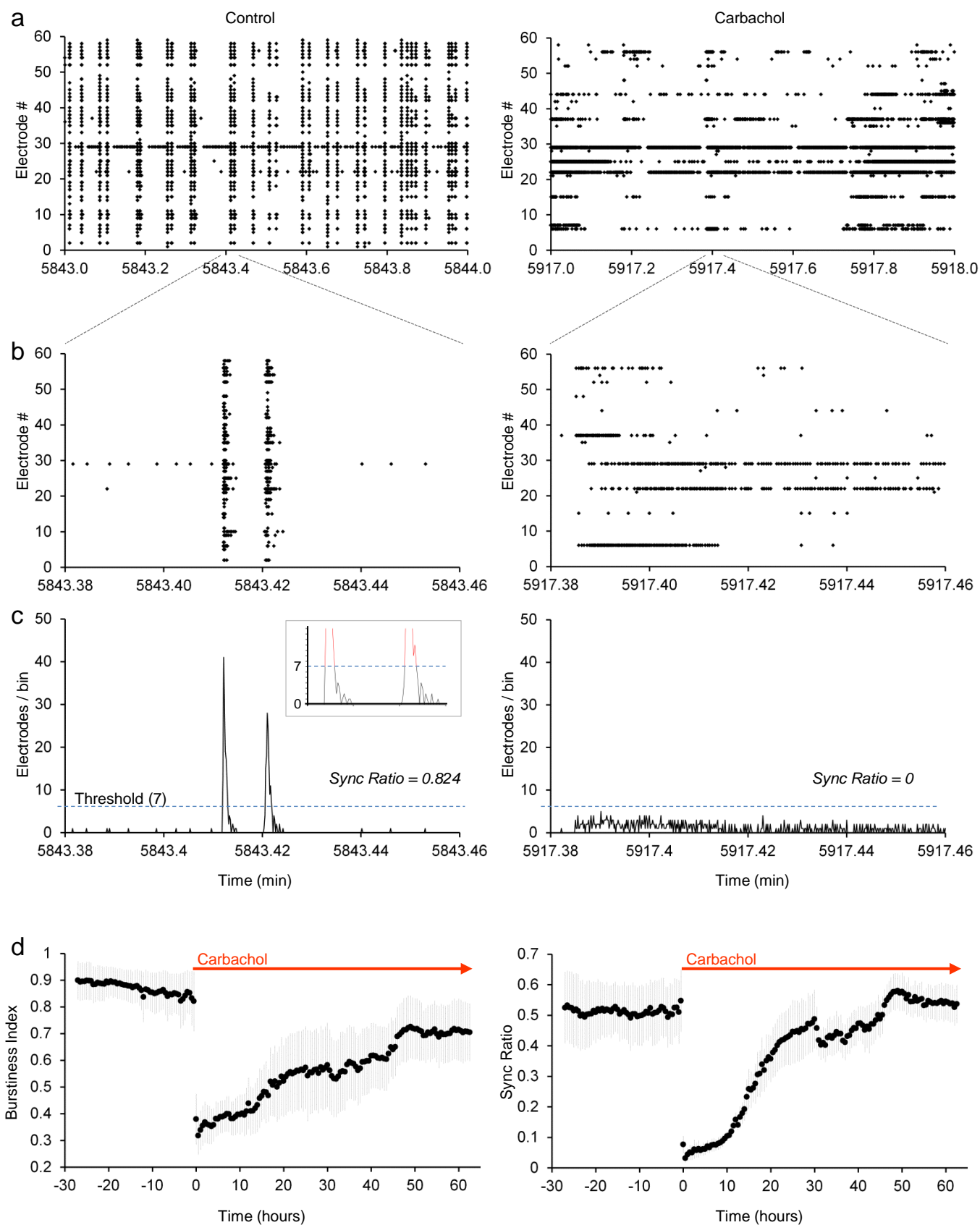

Supplement: Additional file 3: Figure S3. — The Sync Ratio as a measure of network synchrony. The premise of this measure is that synchrony, by definition, implies that many neurons across the network are almost simultaneously active. Therefore, the measure is based on the number of electrodes concomitantly active within a small time window. a) One-minute raster plots of network activity recordings in normal medium (left) and carbachol (CCh; right) showing distinctly different activity patterns. In normal media, activity in all electrodes is highly synchronous, whereas in CCh, activity is much more tonic and asynchronous. Time is given in minutes from the beginning of recording. b) Enlarged portions (4.8 seconds) from each trace in a. c) Active electrode per bin counts (bin =10 mseconds) over 4.8 seconds from each trace shown in b. The dashed line depicts the threshold defined for the Sync Ratio calculation (th =7), clearly discriminating between synchronous events and desynchronized firing. Sync Ratio values denoted were calculated over the 4.8 second data traces. The inset in the left panel shows two synchronous events at a higher magnification, with suprathreshold bins highlighted in red. d) Network synchrony estimated by two measures - Burstiness Index (BI, calculated according to Wagenaar and colleagues [70], with some adjustments of parameters as previously described [35]) and Sync Ratio, calculated at half-hour intervals for previously published experiments [35] in which CCh was used to elevate cholinergic tone. Both measures show the same trends; however, the Sync Ratio, as opposed to the BI, is less affected by increased burst rates near the end of the experiment and fully recovers to pre-CCh levels. [file 12915_2014_83_MOESM3_ESM.pdf]

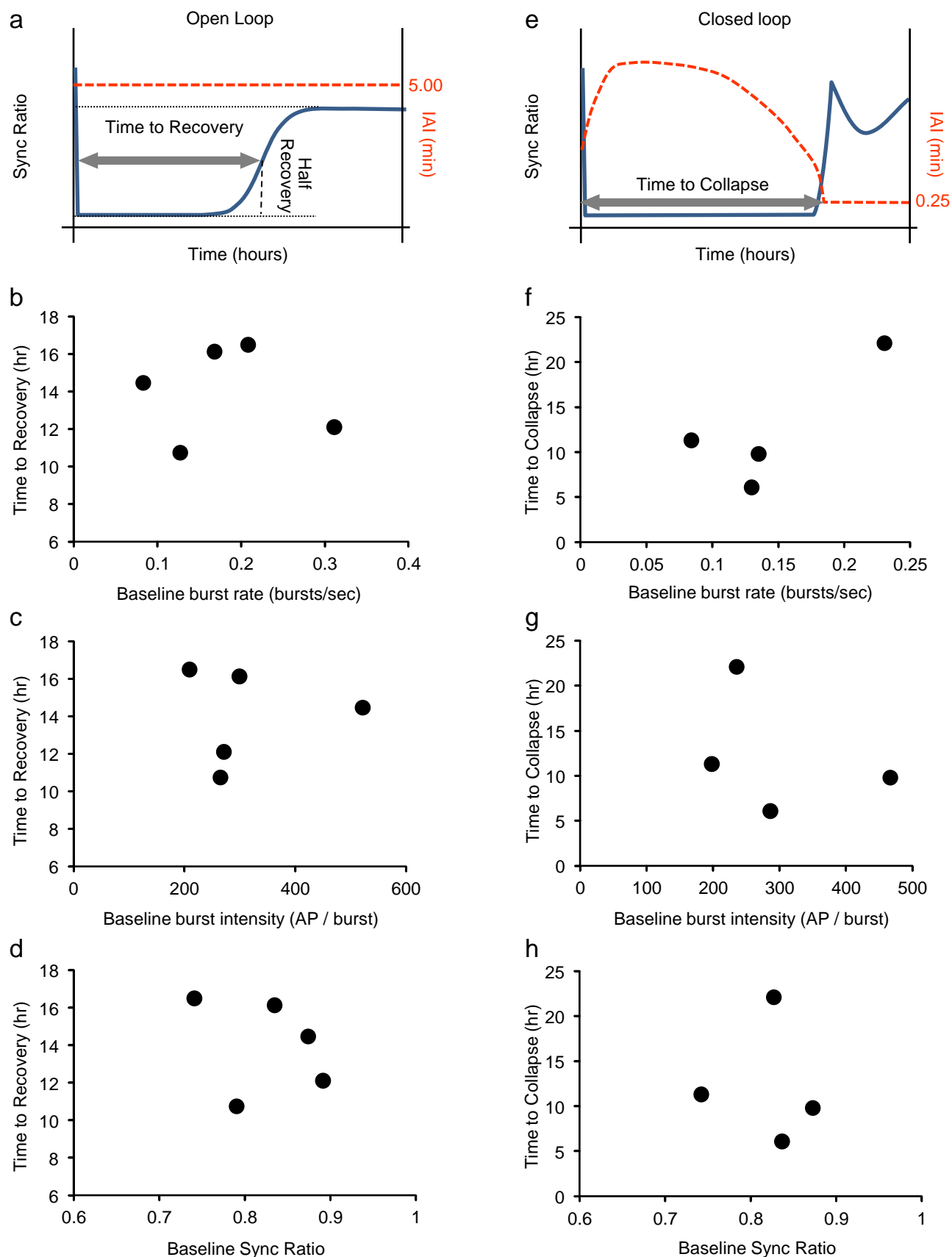

Supplement: Additional file 5: Figure S4. — Synchrony reemergence time is mostly independent of network baseline activity characteristics. a) Illustration of the procedure used to determine the Time to Recovery in the open loop experiments of Figure 3. To that end, Sync ratio values determined at one-minute intervals were smoothed with a five point kernel and the time at which the Sync Ratio exceeded the half recovery point, defined as ≡ min _ val +0.5 ⋅ (max _ val ‐ min _ val) was obtained. No obvious relationships were detected between the Time to Recovery and b) burst rates; c) burst intensities; and d) Sync Ratio values measured during 12 hour baseline recording periods (phase I in Figure 1c) in all open loop experiments of Figure 3. e) Illustration of the procedure used to determine the Time to Collapse in the closed-loop experiments of Figure 4. To that end, the time, in each experiment, at which the inter application interval (IAI) first reached 0.25 minutes was obtained. No obvious relationships were observed between the Time to Collapse and f) burst rates; g) burst intensities; and h) Sync Ratio values measured during 12 hour baseline recording periods (phase I in Figure 1c) in all closed-loop experiments of Figure 4. AP, Action Potentials. [file 12915_2014_83_MOESM5_ESM.pdf]

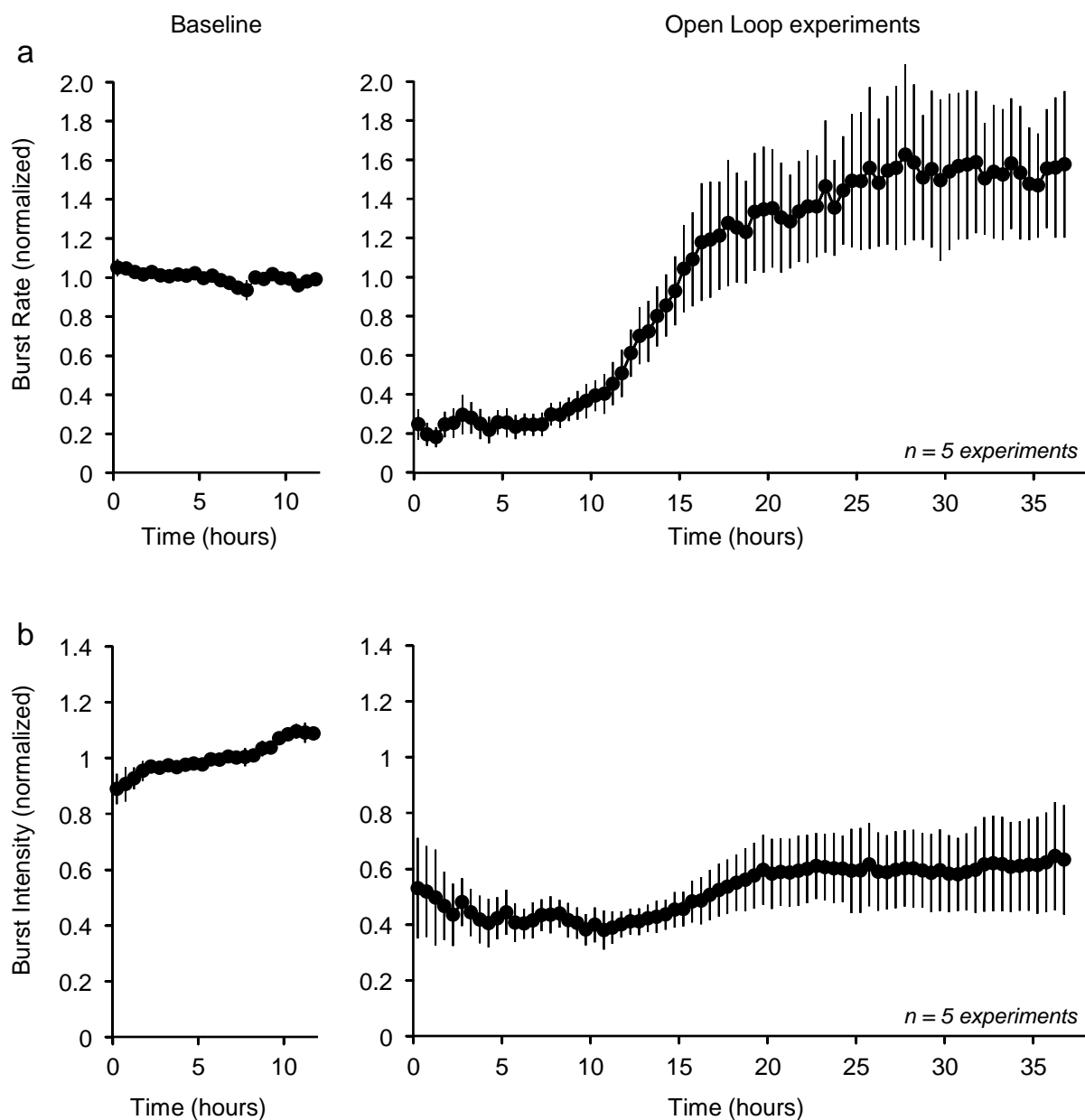

Supplement: Additional file 6: Figure S5. — Changes in burst rates and burst intensities following prolonged ACh applications. a) For each open loop experiment of Figure 3, burst rate values, averaged over 30 minutes, were normalized to the average burst rates measured during 12 hour baseline recording periods (phase I in Figure 1c). Left – baseline periods. Right – open loop ACh application periods (phase V in Figure 1c). b) A similar analysis for burst duration. Averages ± SEM for five experiments. See Methods for further details on burst detection. [file 12915_2014_83_MOESM6_ESM.pdf]

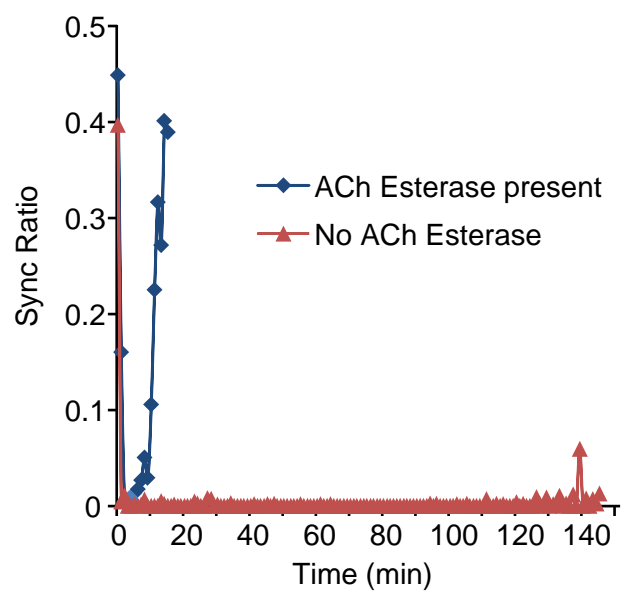

Supplement: Additional file 7: Figure S6. — The presence of AChE is required for phasic-like cholinergic input. The Sync Ratio is depicted over time following a single application of ACh at t =0 with no AChE applied (red) or with AChE in the media (blue; average of two repeats. This AChE concentration (0.1 U/ml) was used in all experiments. [file 12915_2014_83_MOESM7_ESM.pdf]

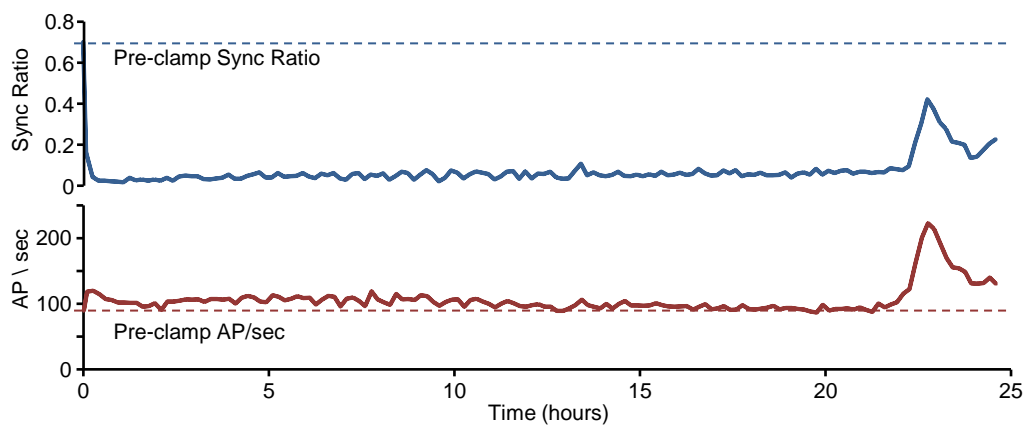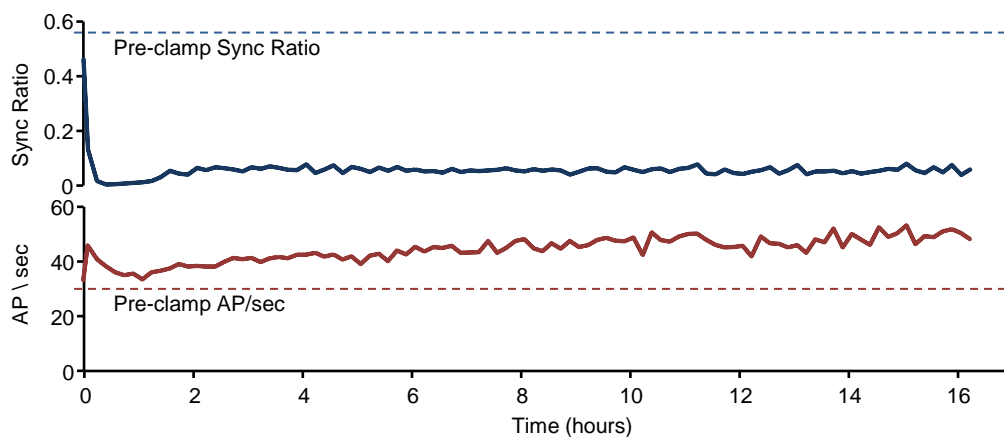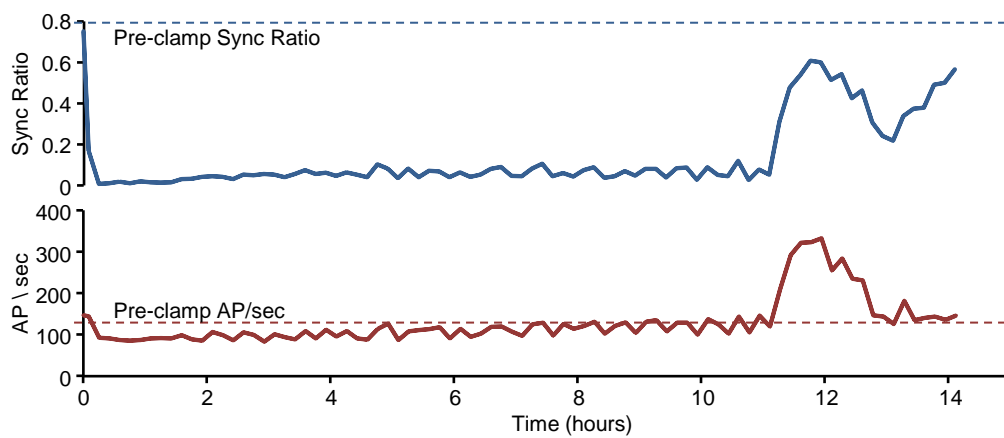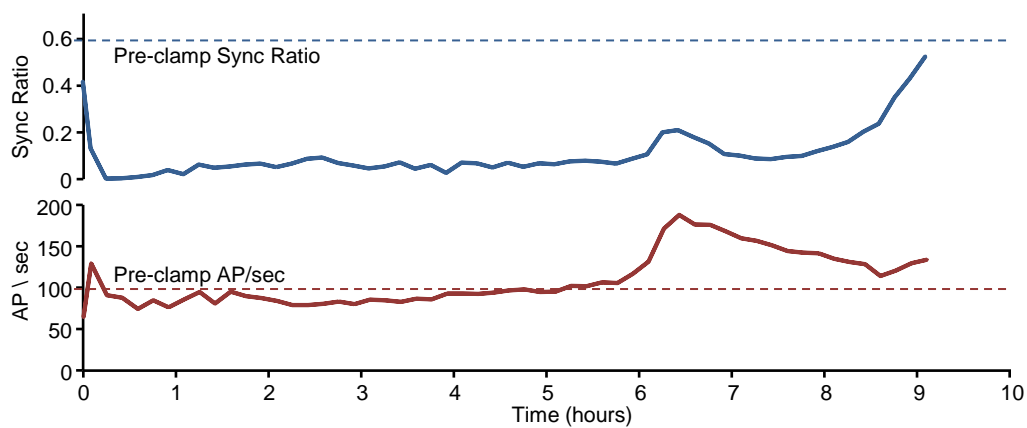

Supplement: Additional file 8: Figure S7. — Comparisons of firing rates and Sync Ratio values in closed-loop experiments. Sync Ratio (blue) and firing rate (red) values, both averaged over 10 minute bins, are shown for each closed-loop experiment shown in Figure 4. In all experiments Sync Ratio values were significantly lower than pre-clamp values, while firing rates were not significantly altered or changed only slowly after the initiation of the clamp (blue and red dashed lines, accordingly). The escape from the clamp was associated with temporary increases in firing rates due to transient periods of volleys of synchronous bursts that were associated with this escape after which firing rates tended to subside while synchrony gradually approached pre-clamp levels (see also Additional file 9: Video S2). AP, Action Potentials. [file 12915_2014_83_MOESM8_ESM.pdf]

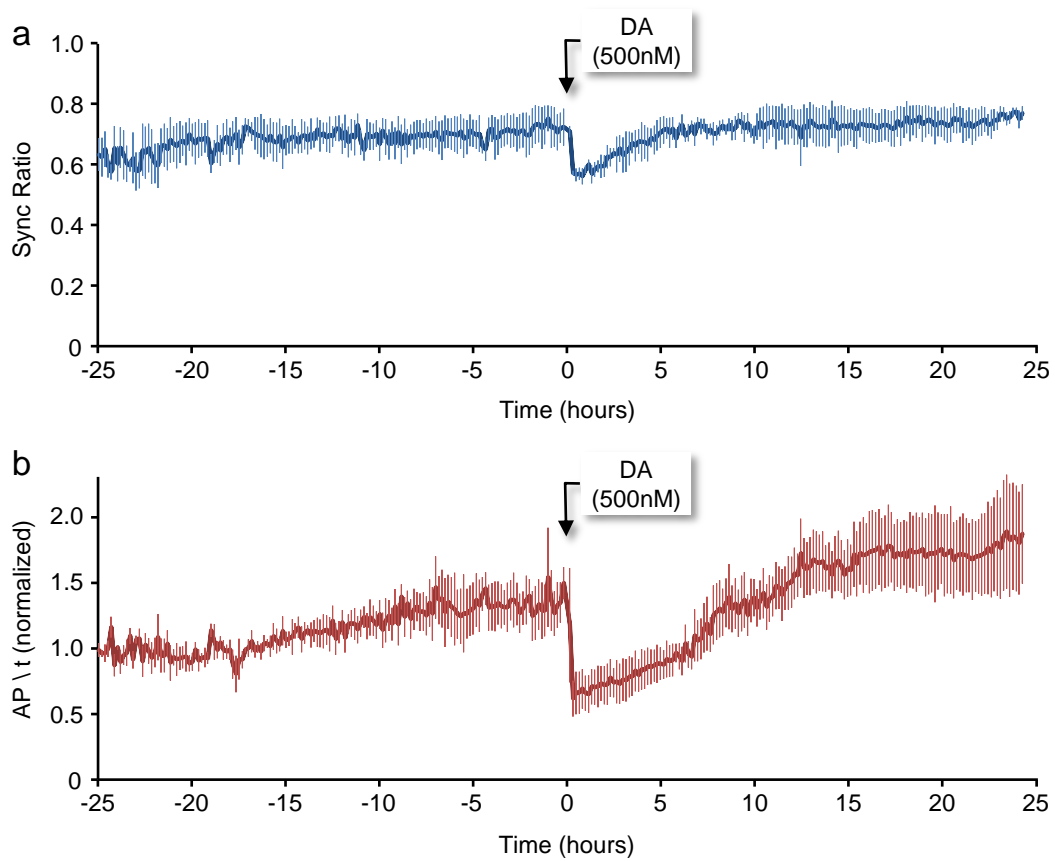

Supplement: Additional file 11: Figure S8. — Dopamine effects on network synchrony and firing rates in networks of cultured cortical neurons. After recording baseline activity for at least 72 hours (of which the last 25 hours are shown), DA (500nM) was added directly into the MEA dishes and the perfusion media reservoirs. The figures show the evolution of the Sync Ratio (a) and normalized firing rates (b) in these experiments, averaged over 10 minute periods. Note the very limited effect of DA on the Sync Ratio, and the more pronounced effect on firing rates. Note also that the Sync Ratio recovered to pre-application levels faster than firing rates did. Averages ± SEM for three separate experiments. AP, Action Potentials. [file 12915_2014_83_MOESM11_ESM.pdf]

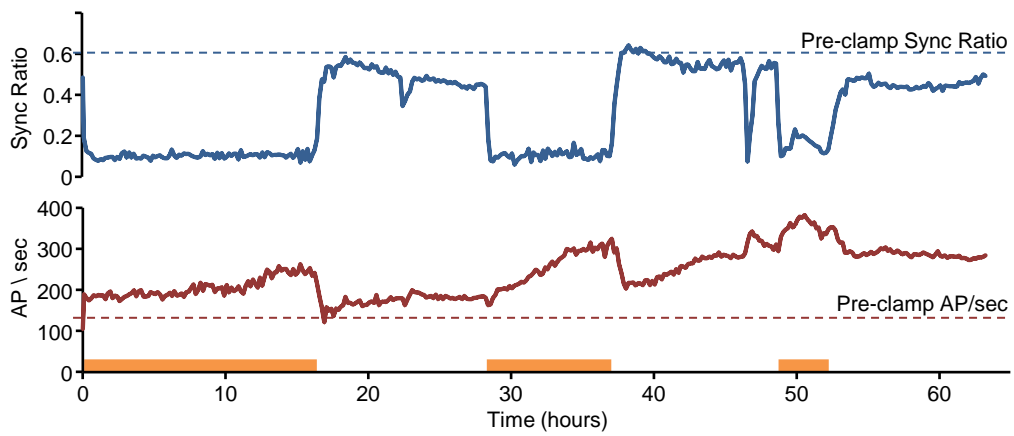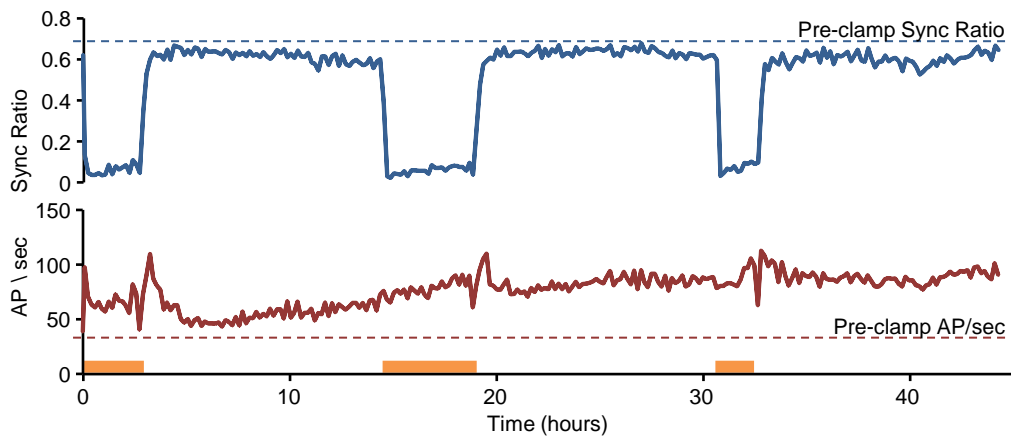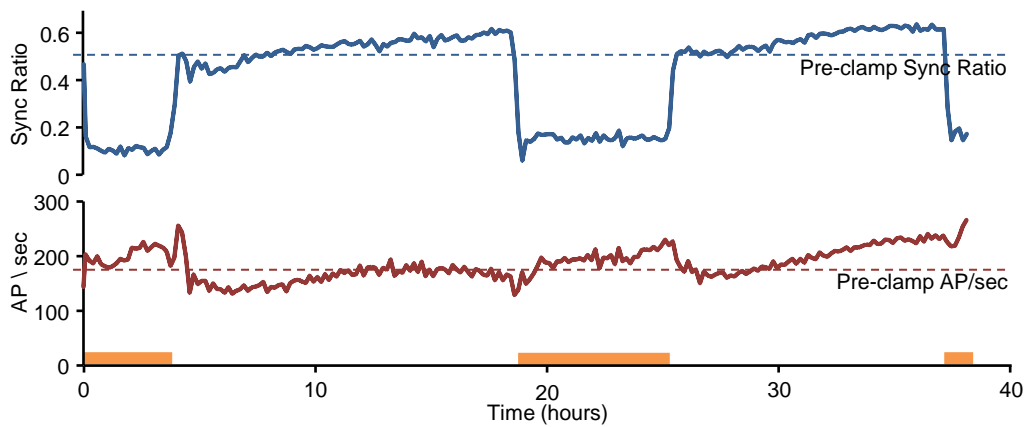

Supplement: Additional file 13: Figure S9. — Firing rates and the Sync Ratio in multi-epoch experiments. Sync Ratio (blue) and firing rate (red) values, both averaged over 10 minute bins, are shown for each multi-epoch experiment shown in Figure 6. Periods in which the synchrony clamp was in effect are shown as thick orange lines. Note that firing rates and Sync Ratio values do not co-vary consistently. AP, Action Potentials. [file 12915_2014_83_MOESM13_ESM.pdf]
